# Associations Between OPN-CD44 Axis Genetic Variability, Plasma Osteopontin, and Treatment Outcomes in Head and Neck Squamous Cell Carcinoma

**DOI:** 10.3390/ijms27093724

**Published:** 2026-04-22

**Authors:** Agnieszka Gdowicz-Kłosok, Regina Deja, Tomasz Rutkowski, Magdalena Bugowska, Jolanta Mrochem-Kwarciak, Krzysztof Składowski, Dorota Butkiewicz

**Affiliations:** 1 Center for Translational Research and Molecular Biology of Cancer, Maria Skłodowska-Curie National Research Institute of Oncology, Gliwice Branch, 44-102 Gliwice, Poland; agnieszka.gdowicz-klosok@gliwice.nio.gov.pl; 2 Analytics and Clinical Biochemistry Department, Maria Skłodowska-Curie National Research Institute of Oncology, Gliwice Branch, 44-102 Gliwice, Poland; 3 Clinical Trials Support Centre, Maria Skłodowska-Curie National Research Institute of Oncology, Gliwice Branch, 44-102 Gliwice, Poland; 4 Department of Biostatistics and Bioinformatics, Maria Skłodowska-Curie National Research Institute of Oncology, Gliwice Branch, 44-102 Gliwice, Poland; 5 Digital Medicine Center, Maria Skłodowska-Curie National Research Institute of Oncology, Gliwice Branch, 44-102 Gliwice, Poland; 6 I Radiation and Clinical Oncology Department, Maria Skłodowska-Curie National Research Institute of Oncology, Gliwice Branch, 44-102 Gliwice, Poland

**Keywords:** osteopontin, OPN/SPP1, CD44, polymorphism, head and neck cancer, treatment response, prognosis, radiotherapy

## Abstract

Inter-individual variability in outcomes following radiotherapy-based treatment remains a major challenge in head and neck squamous cell carcinoma (HNSCC). Osteopontin (OPN) and its receptor CD44 are key mediators of tumor progression, hypoxia-related treatment resistance, and metastatic dissemination. In this exploratory, hypothesis-generating study, we investigated selected functional polymorphisms in *OPN* (*SPP1*) and *CD44* genes, together with pretreatment plasma OPN levels, in relation to overall survival (OS), locoregional recurrence-free survival (LRFS), and metastasis-free survival (MFS) in 242 HNSCC patients treated with curative-intent radiotherapy alone (RT) or combined with chemotherapy (RT + CT). In individual multivariable models, the *OPN* rs11730582 C and *CD44* rs13347 T variants were associated with improved survival outcomes, while elevated OPN levels correlated with shorter OS. In full multivariable models, rs11730582 C and high OPN levels remained independent predictors of OS in the entire cohort. In the RT + CT subgroup, high OPN independently predicted worse OS, whereas rs13347 T was associated with better MFS. In the RT subset, rs11730582 CC independently predicted longer OS. These findings suggest that both germline variability within the OPN-CD44 signaling axis and circulating OPN levels are associated with treatment outcomes in HNSCC patients receiving radiotherapy-based regimens. Given the exploratory design, further validation in independent cohorts is warranted.

## 1. Introduction

Head and neck squamous cell carcinoma (HNSCC) accounts for approximately 5% of all cancers worldwide and is associated with significant and steadily increasing morbidity and mortality. In Europe, the 5-year survival rate for locally advanced HNSCC is only around 50% [1]. Radiotherapy (RT) and chemoradiotherapy (CTRT) remain the primary treatments for HNSCC. However, response to these treatments varies greatly between individuals, often limiting their effectiveness and leading to recurrence and reduced survival rates despite ongoing progress in diagnostics and therapy. Gaining a better understanding of the mechanisms behind treatment resistance and failure is therefore essential for improving clinical outcomes. Consequently, there is increasing interest in identifying markers that can predict recurrence risk and patient survival in HNC. Among these, osteopontin (OPN) has emerged as a promising candidate, both in terms of protein levels in tumors or blood, and in single-nucleotide polymorphisms (SNPs) in the gene encoding OPN.

OPN (first identified as secreted phosphoprotein 1, SPP1) is a pleiotropic phosphorylated glycoprotein expressed in various cell types, including epithelial, endothelial, and immune cells. OPN and its CD44 (cluster of differentiation 44) receptor are key mediators of cell adhesion, migration, epithelial–mesenchymal transition (EMT), metastasis, cancer stemness, angiogenesis, and immune evasion [2,3]. Overexpression of OPN and CD44 is frequently observed in cancers and generally associated with disease aggressiveness, progression, and a poor prognosis in multiple tumor types, including HNSCC [4,5,6]. Furthermore, both OPN and CD44 have been implicated in hypoxia-driven resistance to RT and/or CT [7,8]. High levels of CD44, a marker of cancer stem cells in HNSCC, have been linked to therapy failure, metastasis, and decreased survival in this cancer [9,10,11]. Similarly, elevated circulating and tissue OPN levels have often been associated with adverse treatment response and unfavorable outcomes in HNSCC; however, evidence for their prognostic and predictive value remains inconsistent [12,13].

Germline polymorphisms in the genes encoding OPN and CD44, by influencing protein expression and/or function, may modulate tumor behavior, disease course, and sensitivity to anticancer therapies. In our previous studies, we showed that certain *OPN* (alias *SPP1*) and *CD44* SNPs, as well as high OPN plasma levels, predicted poor outcomes in non-small-cell lung cancer (NSCLC) patients receiving RT or CTRT [14,15]. Although several reports have described an association between *OPN* and *CD44* polymorphisms and cancer susceptibility or prognosis, data on HNSCC are very limited and inconclusive. Therefore, given the involvement of OPN-CD44 signaling in cancer progression, therapy resistance, and immune regulation, the present study aimed to investigate the association of selected functional variants in the *OPN* and *CD44* genes, along with pretreatment OPN plasma levels, with the effects of curative RT and CTRT in HNSCC patients. To our knowledge, this is the first study to jointly evaluate germline variability within the OPN-CD44 axis together with circulating OPN in HNSCC patients treated with RT-based regimens, addressing an important gap in exploratory, hypothesis-generating research on candidate biomarkers in HNSCC.

## 2. Results

### 2.1. Baseline Characteristics

The median OS in the group was 64.9 months, while the median LRFS and MFS were not reached. The median follow-up time since cancer diagnosis was 138.1 months. During observation time, 175 (72%) patients died, 83 (34%) experienced locoregional recurrence, 32 (13%) developed distant metastasis, and 44 (18%) were diagnosed with a second primary cancer (SPC). The genotype distribution for all SNPs in the study group was in accordance with HWE (Supplementary Table S1). The patient characteristics are shown in Table 1 (for description see the Materials and Methods Section).

### 2.2. Clinical Outcomes in Relation to Individual SNPs

**Figure 1:**
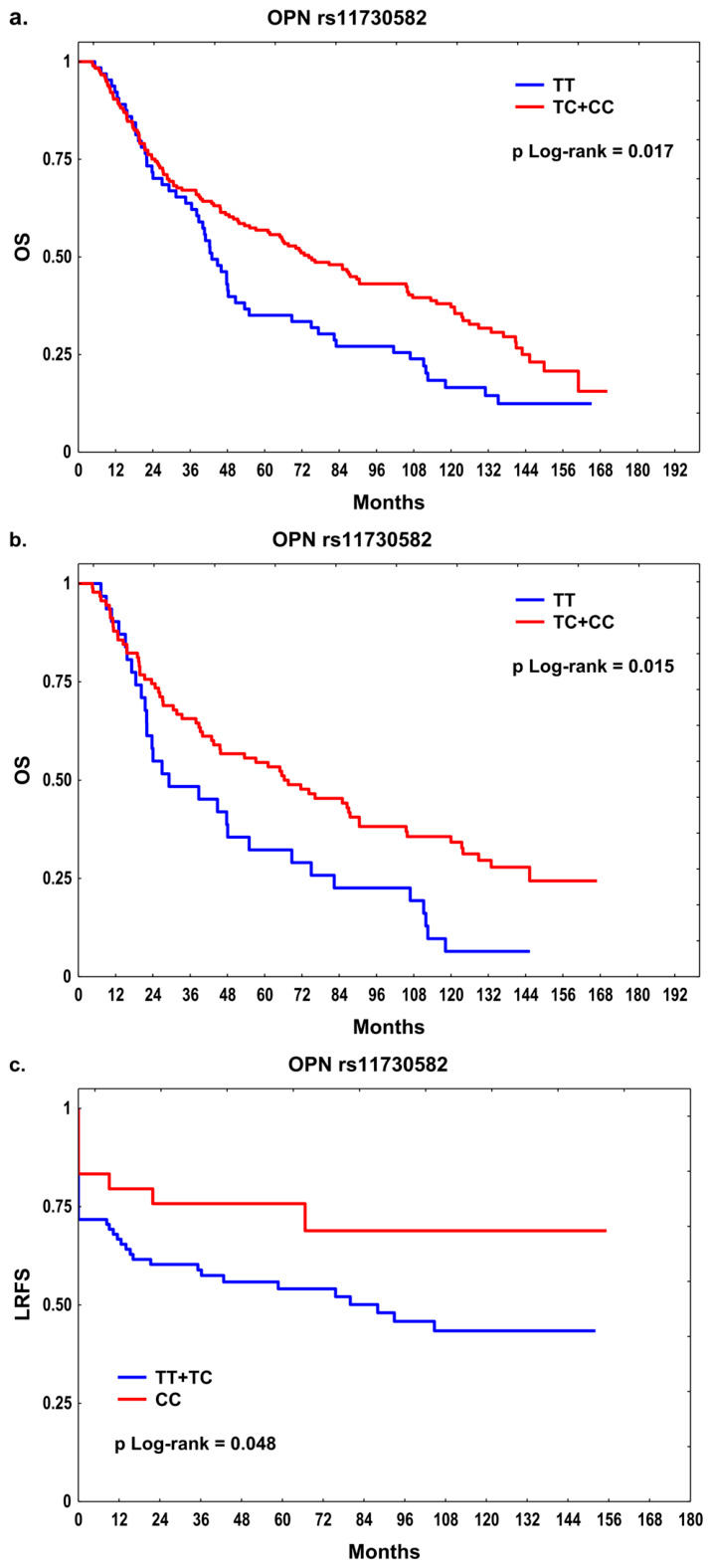
The Kaplan–Meier plots for the *OPN* rs11730582 in relation to (**a**) OS in all patients and (**b**) OS, and (**c**) LRFS in the combination treatment subgroup (RT + CT).

In the entire cohort, carriers of the *OPN* rs11730582 C allele showed significantly longer OS than TT homozygotes (72.7 vs. 42.4 months, *p* = 0.017; Figure 1a). In both univariable and multivariable analyses adjusted for clinical covariates, the rs11730582 C allele was associated with improved OS (HR = 0.66, 95% CI: 0.48–0.92, *p* = 0.013 and HR = 0.67, 95% CI: 0.48–0.93, *p* = 0.017, respectively), whereas the *CD44* rs13347 T allele was associated with longer MFS only in the multivariable model (HR = 0.41, 95% CI: 0.17–0.99, *p* = 0.047; Table 2).

Patients were then stratified by treatment to better evaluate the associations between SNPs and therapy outcomes within clinically more homogeneous subgroups. In the combination treatment subgroup (RT + CT), carriers of the *OPN* rs11730582 C allele demonstrated significantly longer OS compared with TT homozygotes (65.5 vs. 27.0 months, *p* = 0.015; Figure 1b). Moreover, CC homozygotes showed better LRFS compared with T variant carriers (median not reached vs. 80.0 months, *p* = 0.048; Figure 1c). The *CD44* rs13347 T allele carriers had better MFS than CC homozygotes, but the difference did not reach statistical significance (*p* = 0.061). Multivariable analysis showed that the presence of the rs11730582 C allele was significantly associated with a lower risk of death (HR = 0.60, 95% CI: 0.38–0.96, *p* = 0.032), while the CC genotype significantly reduced the risk of recurrence after RT + CT (HR = 0.44, 95% CI: 0.20–0.96, *p* = 0.040). The *CD44* rs13347 T allele was associated with a decreased risk of distant relapse (HR = 0.23, 95% CI: 0.07–0.70, *p* = 0.010) in the RT + CT subset (Table 2). In the subgroup treated with RT alone, no significant associations with clinical outcomes were found.

After controlling for the false discovery rate (FDR), the relationship between rs11730582 and OS in univariate models remained statistically significant (Supplementary Table S2).

### 2.3. Haplotype Analysis

Linkage disequilibrium (LD) analysis showed high D′ values (>0.8) between *OPN* rs11730582 and rs1126772 (D′ = 0.86, 95% CI: 0.79–0.92), *OPN* rs4754 and rs1126772 (D′ = 0.97, 95% CI: 0.94–1.00), and *CD44* rs7116432 and rs13347 (D′ = 0.82, 95% CI: 0.74–0.89) (Supplementary Figure S1). However, the corresponding r^2^ values were low to moderate (0.21, 0.63 and 0.11, respectively), indicating incomplete correlation between these loci and suggesting that they provide non-redundant genetic information. Therefore, haplotype analysis was conducted as an exploratory approach, while individual SNPs were retained in the final models. For *OPN*, the rs11730582-rs4754-rs1126772 haplotypes had frequencies of 44% (T-T-A), 28% (C-T-A), 18.3% (C-C-G), 7% (T-C-A), and 1.5% (T-C-G), whereas the *CD44* rs7116432-rs13347 haplotypes occurred with frequencies of 41.1% (G-C), 40.1% (A-C), 17.3% (A-T), and 1.5% (G-T). Haplotypes with frequencies below 1% were excluded from further analyses.

In the overall cohort, selected *OPN* haplotypes were associated with survival outcomes. The C-T-A haplotype was associated with improved OS, whereas T-T-A and T-C-G haplotypes were linked to worse outcomes. In multivariable analysis, the C-T-A haplotype was associated with reduced mortality risk (HR = 0.61, *p* = 0.002), while T-T-A was associated with an increased risk of death and locoregional failure (HR = 1.56, *p* = 0.024 and HR = 1.85, *p* = 0.026, respectively). In the RT + CT subgroup, the T-T-A haplotype correlated with shorter LRFS (*p* = 0.017) and increased risk of locoregional recurrence (HR = 2.41, *p* = 0.013), whereas the *OPN* C-T-A and *CD44* A-T haplotypes were linked to improved OS and MFS, respectively. In the RT-only subgroup, only the C-T-A haplotype remained associated with better OS. Detailed results are presented in Supplementary Table S3 and Figure S2.

### 2.4. Plasma OPN Levels and Survival Outcomes

**Figure 2:**
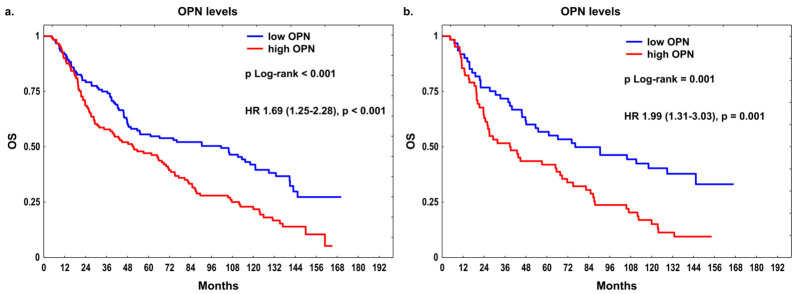
Overall survival (OS) in relation to the OPN pretreatment levels in plasma (**a**) in the whole group and (**b**) in the combination treatment subgroup (RT + CT).

The mean ± SD OPN levels were 61.2 ± 45.4 ng/mL in the entire cohort, 58.9 ± 39.7 ng/mL in the RT-only subgroup, and 63.4 ± 50.4 ng/mL in the RT + CT subgroup. The median OPN levels were 59.8 (Q1–Q3: 15.4–89.3) in all patients, 62.4 (Q1–Q3: 16.1–84.6) in the RT-only subgroup, and 56.9 (Q1–Q3: 15.4–102.8) in the RT + CT subgroup. Patients with positive lymph nodes (N > 0) had higher median OPN concentration in plasma than those with N0, both in the entire group (67.5 vs. 51.5 ng/mL, respectively, *U* = 5647.5, *p* = 0.018) and in the RT-only subset (71.5 vs. 56.5 ng/mL, respectively, *U* = 1254.5, *p* = 0.030). No other significant associations were observed between OPN levels and clinico-demographic factors. Moreover, no significant associations of the studied *OPN* SNPs with pretreatment circulating OPN levels were found in the entire cohort and treatment subgroups (Supplementary Table S4). When patients were stratified by median OPN levels, individuals with high OPN concentration had significantly shorter OS than those with low OPN levels in the whole cohort (51.3 vs. 101.5 months, *p* < 0.001) and in the RT + CT subset (39.3 vs. 76.2 months, *p* = 0.001) (Figure 2a,b). High OPN levels were strongly associated with an increased risk of death in univariable analysis in all patients (HR = 1.69, 95% CI: 1.25–2.28, *p* < 0.001) and in the RT + CT subgroup (HR = 1.99, 95% CI: 1.31–3.03, *p* = 0.001). The association between OPN levels and OS survived the FDR adjustment for multiple comparisons only in univariable analysis (Supplementary Table S2). In multivariable models adjusted for clinical confounders (Table 2), high OPN concentration remained significantly associated with poor OS in the entire cohort (HR = 1.54, 95% CI: 1.12–2.12, *p* = 0.008) and in the RT + CT subset (HR = 1.66, 95% CI: 1.04–2.66, *p* = 0.035). However, in the RT-only subgroup, this association was only borderline significant (*p* = 0.061).

### 2.5. Integrated Analysis of Molecular and Clinical Factors for OS, LRFS and MFS

To identify independent prognostic factors, all molecular and clinico-demographic factors were simultaneously included in multivariable models for each endpoint. The resulting full models are presented in Table 3. In the entire cohort, both the *OPN* rs11730582 SNP and pretreatment plasma OPN levels remained significantly associated with OS. Carriers of the rs11730582 C allele had a reduced risk of mortality (HR = 0.61, 95% CI: 0.41–0.91, *p* = 0.015), while high OPN levels were associated with an increased risk of death (HR = 1.62, 95% CI: 1.16–2.26, *p* = 0.004), identifying both factors as independent predictors of OS. No SNPs or OPN levels were independently associated with LRFS, whereas a trend towards improved MFS was observed for patients carrying the *CD44* rs13347 T variant (*p* = 0.088).

In the RT + CT subgroup, high OPN levels remained independently associated with worse OS (HR = 1.93, 95% CI: 1.15–3.23, *p* = 0.013). In addition, the *CD44* rs13347 T allele was identified as an independent indicator of improved MFS (HR = 0.19, 95% CI: 0.06–0.66, *p* = 0.009). The *OPN* rs11730582 SNP showed a trend towards association with OS and LRFS, but these did not reach statistical significance in the full model (*p* = 0.076 and 0.078, respectively). In patients treated with RT alone, the *OPN* rs11730582 CC genotype was independently associated with OS (HR = 0.43, 95% CI: 0.21–0.89, *p* = 0.023). No significant associations were observed for LRFS, and the model for MFS could not be reliably constructed due to the limited number of events.

FDR-adjusted results are provided in Supplementary Table S5. After correction, none of the observed associations retained statistical significance, indicating that these findings should be interpreted as exploratory and hypothesis-generating, warranting independent validation.

## 3. Discussion

OPN-CD44 signaling has been proposed as a pathway involved in tumor progression and treatment resistance, particularly in the context of hypoxia. In this study, we investigated the potential prognostic significance of selected genetic polymorphisms in the *OPN* and *CD44* genes, as well as pretreatment circulating OPN levels, in Caucasian patients with HNSCC who received curative radiotherapy with or without cisplatin-based chemotherapy. Our findings indicate that two common germline variants—rs11730582 T>C in the *OPN* promoter and rs13347 C>T in the *CD44* 3′ untranslated region (3′UTR)—emerged as independent predictors of treatment outcomes, with elevated baseline OPN plasma concentration associated with poor OS. Notably, these effects varied according to treatment modality. Importantly, these observations should be interpreted as clinical associations rather than evidence of direct functional mechanisms.

Interestingly, the *OPN* rs11730582 C allele was associated with improved OS in the entire cohort, while the CC genotype showed a significant association with OS specifically in the RT-only subgroup. In addition, exploratory haplotype analysis suggested potential combined effects of the *OPN* variants (e.g., the C-T-A haplotype appeared associated with longer OS, while T-T-A was linked to inferior OS and LRFS); however, these were not included in the final model due to weak LD and should be interpreted as supportive, hypothesis-generating evidence. These findings are in agreement with those in hepatocellular carcinoma, where carriers of the T allele and related haplotypes demonstrated shorter OS and time to recurrence [16]. The presence of the C allele has also been shown to reduce susceptibility to oral and nasopharyngeal cancers [17,18], as well as breast cancer and glioma [19,20]. In contrast, studies in gastric and lung cancers [21,22,23]—including our previous work in NSCLC treated with RT and CTRT [15]—have linked the C allele and the CC genotype to unfavorable treatment responses and survival. The rs11730582 resides within the gene promoter and alters transcription factor binding, such as c-Myb [24,25]. Functional consequences of this SNP remain unclear. Dong et al. [16] showed that the TT genotype increased promoter activity, protein production, and the proliferation and metastasis of hepatocellular carcinoma cells. At the same time, other authors found that C allele enhanced transcriptional activity in melanoma and gastric cancer [21,24]. In our HNSCC cohort, the C variant was protective, suggesting a potential association with OPN-related signaling in the tumor microenvironment rather than demonstrating a direct effect on systemic protein levels. From a biological perspective, one may speculate that the C allele could be linked to alterations in OPN-related processes (e.g., tumor cell migration, invasion, angiogenesis, and immune response), which could contribute to differences in therapeutic sensitivity and patient survival. However, this interpretation remains hypothetical and requires experimental validation. Unfortunately, direct evidence for the functional effects of this SNP in HNC is still missing. To date, only Wang et al. [18] have reported lower serum OPN concentrations in C allele carriers with nasopharyngeal carcinoma; however, we found no association between plasma OPN levels and *OPN* genotypes in our cohort. This reinforces that the prognostic effect of rs11730582 likely operates independently of systemic OPN levels. Taken together, these observations (including our own earlier results in NSCLC [15]) suggest that the phenotypic consequences and biological significance of *OPN* polymorphisms may vary across cancer types, likely due to differences in tissue-specific gene regulation, tumor biology, and microenvironmental characteristics. Additional research in HNSCC is needed to clarify how this SNP modulates OPN expression and function in a disease-specific manner.

Another independent association was observed between the *CD44* rs13347 T allele and a lower risk of distant metastasis after combination therapy. Haplotype analysis (rs7116432-rs13347 A-T) provided additional supportive evidence. Our results confirm findings in colon cancer, where the T variant conferred a protective effect on OS [26], but contrast with a breast cancer study, in which the T allele predicted a lower survival rate [27]. To date, these are the only two published studies on the prognostic impact of this SNP in cancer. The *CD44* rs13347 T has also been identified as a risk factor for various malignancies, including nasopharyngeal carcinoma, primarily in the Asian population [27,28,29]. The rs13347 C>T SNP, located in the 3′UTR, may influence gene expression and mRNA stability. CD44 expression is tightly regulated post-transcriptionally by multiple microRNAs (miRNAs) that modulate its role in cell adhesion, migration, and stemness. Functional studies indicate that this substitution disrupts the miR-509-3p binding site, resulting in reduced miRNA-mediated repression and increased reporter gene activity for the T allele. Furthermore, carriers of the T variant exhibited elevated CD44 protein levels [27,29,30]. These data suggest that further research is necessary to elucidate the functional role of rs13347 and miR-509-3p regulation in HNSCC. Nevertheless, the observed association of the T allele with lower metastasis risk in the combination therapy subset may reflect context-dependent clinical associations potentially related to CD44 biology rather than a direct causal relationship, possibly involving altered expression of metastasis-promoting CD44 isoforms, or modified interactions with the tumor microenvironment. Our results reinforce existing evidence linking CD44 (and its genetic variation) to cancer progression and metastatic potential in solid tumors [3].

We found that elevated plasma OPN levels before treatment were strongly related to shorter OS both in the whole cohort and in the combination therapy subgroup. Baseline circulating OPN concentrations represent both the tumor and host microenvironment, and may serve as a systemic indicator of disease activity and burden. Higher plasma OPN may indicate increased tumor secretion and host stromal activation, suggesting a more hypoxic, aggressive tumor biology, a more infiltrative phenotype, or an immunosuppressive milieu. Numerous studies have evaluated OPN as a prognostic marker in HNC, but their results vary, partly due to heterogeneity among patient cohorts, analytical assays, sample types and timing, and treatment modalities. While most of them report that elevated plasma OPN levels correlate with adverse outcomes, a large phase III HNSCC trial did not confirm this association [13]. However, a recent meta-analysis of 51 studies demonstrated that high plasma and tissue OPN levels were linked to worse survival, advanced stage, and disease progression in HNC [12]. OPN can be induced by hypoxia and is part of hypoxia-regulated pathways [2,31]. In HNSCC, plasma OPN has been shown to correlate with tumor hypoxia measures (e.g., pO_2_) and has been proposed as a minimally invasive hypoxia surrogate [32,33,34]. Overgaard et al. [34], for example, suggested that elevated OPN levels predict benefit from hypoxia-modifying therapy with the radiosensitizer nimorazole and may aid in patient selection for this treatment. Conversely, Lim et al. [13] failed to demonstrate that plasma OPN predicts response to hypoxia-targeting therapy using tirapazamine. In summary, our findings align with most existing research, reinforcing the potential value of plasma OPN levels as a systemic indicator of adverse outcomes in HNSCC treated with RT-based regimens. Additionally, since tumor hypoxia contributes to increased tumor aggressiveness, therapy resistance, and poor outcomes, our observations are consistent with a potential role of OPN as a clinical marker associated with hypoxia and radioresistance in HNSCC.

Furthermore, we observed no correlation between the examined *OPN* genotypes and plasma OPN concentration, which may reflect the complex regulation of circulating OPN levels and the potentially stronger influence of tumor-related factors. Consistent with other studies, we have previously reported similar results in NSCLC [15,35]. These observations suggest that the prognostic effect of the rs11730582 may operate independently of circulating OPN levels, although functional and mechanistic studies are needed. For example, germline genetic variants could affect OPN function, its cellular localization, and/or receptor interactions, rather than influencing systemic protein levels. The independent associations observed in the multivariable analysis highlight the relevance of rs11730582, rs13347, and plasma OPN as subset-specific prognostic factors. Chemotherapy and radiotherapy can induce OPN upregulation in tumor cells and the surrounding microenvironment as part of the stress response and tissue repair processes. Recent studies suggest that OPN inhibition may represent a potential therapeutic strategy to enhance radiosensitivity and overcome treatment resistance [36,37,38]. Various approaches targeting OPN are currently under investigation, including monoclonal antibodies, small-molecule inhibitors, aptamers, and small interfering RNAs [2,31,39]. Taken together, our findings suggest a potential clinical relevance of components of the OPN-CD44 signaling axis in HNSCC biology, while underscoring the need for cautious interpretation given the exploratory nature of our study.

Several limitations of our study should be acknowledged. First, although our cohort represents one of the larger single-center series in this research area, the sample size may have limited the statistical power to detect modest effects typical of SNPs, particularly within treatment subgroups. Second, only a selected panel of SNPs and genes was evaluated, while additional genetic variants and molecular pathways may also influence treatment response and clinical outcomes in HNSCC. Third, tumor expression of OPN and CD44 was not assessed, which prevented direct analyses of genotype–phenotype relationships at the tissue level. Fourth, an additional limitation is the lack of HPV/p16 status, a key determinant of biological heterogeneity and prognosis in HNSCC, particularly in OPSCC. Tumor location was not used as a surrogate for HPV status in this study, and no assumptions regarding HPV positivity were made. However, the absence of HPV/p16 data, due to the retrospective nature of the cohort, precluded analyses adjusted for this factor and may introduce residual confounding. Future studies should consider HPV/p16 status to better account for this important source of heterogeneity. Fifth, mechanistic evidence regarding the functional impact of rs11730582 and rs13347 in HNSCC remains limited; therefore, the observed associations should be interpreted cautiously despite their biological plausibility. Therefore, no causal or functional conclusions can be drawn from this study. Sixth, our study group was drawn from a single ethnic population, which may restrict the generalizability of our findings. Finally, given the exploratory, hypothesis-generating design of this discovery-stage study and the moderate cohort size, the results should be interpreted with caution. Although adjustment for multiple testing was applied as a sensitivity analysis, some observed associations may still be susceptible to false-positive findings. Future studies in larger cohorts, integrating comprehensive genomic profiling, tumor expression analyses, and functional validation, are required to confirm these associations and to further elucidate the underlying biological mechanisms. In particular, such investigations should aim to characterize downstream molecular pathways, including receptor interactions and microenvironment context, to determine whether these associations reflect causal biological processes and contribute to variability in therapy outcomes in HNSCC.

## 4. Materials and Methods

### 4.1. Patients

The study included a total of 242 European Caucasian patients with primary T1-4N0-3M0 head and neck squamous cell carcinoma (HNSCC) and a WHO performance status of 0–1, scheduled for curative treatment at the Maria Skłodowska-Curie National Research Institute of Oncology in Gliwice, Poland. Patients with distant metastases at diagnosis (M1), recurrent disease, or those who had previously been treated for other malignancies were excluded. The mean age at diagnosis ± standard deviation (SD) was 58.2 ± 9.6 years (range 19–80), while the median age at diagnosis was 59 years (53–64). Most patients had locally advanced stage III–IV disease (*n* = 177, 73%) and a history of cigarette smoking (78%). The most common primary tumor sites were the larynx (*n* = 91, 38%) and oropharynx (*n* = 95, 39%) (Table 1). Details regarding treatment and follow-up were described in our previous study [40]. Briefly, patients received radical radiotherapy alone (RT; *n* = 119, 49%) or in combination with cisplatin-based chemotherapy (RT + CT; *n* = 123, 51%) administered as an induction CT and/or concomitantly with RT. The median total radiation dose was 70 Gy (range: 50–75 Gy). Clinical data were collected from medical records.

### 4.2. OPN and CD44 SNP Identification

Six common SNPs were examined in this study, including *OPN* rs1126772, rs11730582, rs4754, *CD44* rs187116, rs13347, and rs7116432 (Supplementary Table S1). The following selection criteria were used: variants exhibited a minor allele frequency (MAF) of ≥20% in the European Caucasian population and a linkage disequilibrium (LD) measured with the squared correlation coefficient r^2^ threshold of <0.8 (The 1000 Genomes project phase 3, www.ensembl.org, https://ldlink.nih.gov accessed on 28 February 2022), had been associated with cancer, and/or had documented or potential functional significance, and/or were located in regulatory or coding regions or domains important for protein activity. Genomic DNA was extracted from frozen peripheral blood using the Genomic Maxi AX kit (A&A Biotechnology, Gdynia, Poland). SNP identification was performed using commercially available Taqman SNP Genotyping Assays (i.e., C_1840808_10, C_7619022_10, and C_27142828_10; Applied Biosystems, Foster City, CA, USA), following the manufacturer’s protocol, or the polymerase chain reaction–restriction fragment length polymorphism (PCR-RFLP) method as described previously [15]. Genotyping was repeated in 10% of randomly selected samples, achieving 100% concordance.

### 4.3. Measurement of Plasma OPN Concentration

Peripheral blood samples were collected before treatment and processed as previously described [14]. Plasma samples were stored at −80 °C until analysis. Circulating OPN levels were quantified using an enzyme-linked immunosorbent assay (ELISA) Human OPN Quantikine ELISA kit, DOST00 (R&D Systems Inc., Minneapolis, MN, USA) and a plate reader Victor 1420 Multilabel Counter (Wallac/Perkin Elmer Life Sciences, Turku, Finland), according to the manufacturer’s instructions. All samples were run in duplicate and the mean value was used for analysis.

### 4.4. Statistical Analysis

The study endpoints were overall survival (OS), locoregional recurrence-free survival (LRFS), and metastasis-free survival (MFS). OS was measured from the date of diagnosis until death from any cause or the last confirmed date the patient was alive. LRFS and MFS were calculated from the last day of treatment to the date of clinically detectable relapse or the last examination showing no evidence of disease. Survival curves were generated using the Kaplan–Meier method and compared with the log-rank test. Hazard ratios (HRs) and 95% confidence intervals (CIs) were estimated using univariable and multivariable Cox proportional hazards regression models. To identify independent prognostic factors while accounting for a moderate sample size and potential correlations between variables, a two-stage multivariable approach was adopted. In the first stage (individual adjusted models), each SNP and OPN protein levels were evaluated in separate multivariable models adjusted for a set of clinico-demographic covariates selected a priori: age at diagnosis, sex, T stage, N stage, primary tumor subsite, chemotherapy use, and smoking/alcohol status. In the second stage, a final full multivariable model was constructed for each endpoint, including all variables simultaneously. No variable selection procedure was applied in the final model. In cases where the full model failed to converge due to a limited number of events, results from the individual adjusted models were reported. As a sensitivity analysis, *p*-values were additionally adjusted for multiple testing using the Benjamini–Hochberg false discovery rate (FDR) method, and are reported in the Supplementary Materials. Given the exploratory nature of the study and the moderate sample size, all primary conclusions were based on uncorrected *p*-values. All SNPs were evaluated under additive, dominant and recessive genetic models, and the most appropriate model was selected based on consistency of effect estimates and model fit. Pearson’s chi-square (χ^2^) test assessed associations between variables and tested for deviations from Hardy–Weinberg Equilibrium (HWE). Continuous variables were reported as means ± standard deviations (SD) for normally distributed data or medians with interquartile ranges for non-normal data, with normality assessed via the Shapiro–Wilk test. Categorical variables are presented as counts and percentages. OPN levels were compared between two groups using the Mann–Whitney *U* test and among three groups using the Kruskal–Wallis H test. Post hoc comparisons were omitted if no significant differences were observed. Linkage disequilibrium (LD) between SNPs was measured using both Lewontin’s deviation coefficient (D’) and r^2^. As the observed r^2^ values were below the commonly accepted threshold for proxy variants (<0.8), indicating incomplete correlation between loci, each SNP was analyzed individually to preserve locus-specific information, and all SNPs were retained in the multivariable model. Haplotypes and their frequencies were estimated using PHASE v2.1.1 [41]. The recombination model implemented in PHASE was applied for haplotype reconstruction. The algorithm was run 10 times with 100 iterations each to improve uncertainty estimates. All statistical tests were two-sided and *p* ≤ 0.05 was considered statistically significant. Statistical analyses were performed in the R environment (v4.4.1, “Race for Your Life”; The R Foundation for Statistical Computing; https://www.r-project.org accessed on 22 October 2024), and STATISTICA 13.1 (TIBCO Software Inc., Palo Alto, CA, USA).

## 5. Conclusions

Our findings suggest that germline variability within genes related to the OPN-CD44 signaling pathway (specifically rs11730582 and rs13347), together with pretreatment plasma OPN levels, may be associated with differences in treatment outcomes in HNSCC patients receiving RT-based regimens. These observations generate testable hypotheses regarding the role of host genetics in modulating treatment response. However, at present, the identified variants should be regarded as statistical biomarkers of prognosis rather than established functional drivers in HNSCC. Further external validation in independent cohorts, along with mechanistic investigations, is required to confirm the reproducibility, biological significance, and potential clinical relevance of these associations.

## Figures and Tables

**Table 1:** Demographic and clinical characteristics of the patients.

Feature	All Patients (*n* = 242)	RT + CT Subgroup (*n* = 123)	RT-Only Subgroup (*n* = 119)
Median age at diagnosis, *n* (%) <59 years ≥59 years	119 (49) 123 (51)	74 (60) 49 (40)	45 (38) 74 (62)
Gender, n (%) Male Female	193 (80) 49 (20)	100 (81) 23 (19)	93 (78) 26 (22)
Primary tumor location, *n* (%) Oropharynx (OPSCC) Hypopharynx (HPSCC) Larynx (LSCC) Nasopharynx (NPSCC) Oral cavity (OSCC)	95 (39) 32 (13) 91 (38) 20 (8) 4 (2)	59 (48) 23 (19) 21 (17) 20 (16) 0 (0)	36 (30) 9 (7) 70 (59) 0 (0) 4 (3)
T stage, *n* (%) 1–2 3–4	122 (50) 120 (50)	36 (29) 87 (71)	86 (72) 33 (28)
N stage, *n* (%) 0 1–3	92 (38) 150 (62)	17 (14) 106 (86)	75 (63) 44 (37)
Smoking status, *n* (%) Never Ever	54 (22) 188 (78)	31 (25) 92 (75)	23 (19) 96 (81)
Alcohol consumption, *n* (%) Never Ever	57 (24) 185 (76)	27 (22) 96 (78)	30 (25) 89 (75)

RT, radiotherapy; CT, chemotherapy.

**Table 2:** Individual SNPs and OPN levels in relation to OS, LRFS and MFS in all HNSCC patients and treatment subgroups.

Endpoint	Variable	Category	Events/*n* ^a^	uHR (95% CI)	*p*	aHR (95% CI) ^b^	*p*
All patients (*n* = 242)							
OS	*OPN* rs4754	TT TC + CC	92/128 83/114	1 1.04 (0.77–1.41)	0.756	1 1.10 (0.80–1.50)	0.559
	*OPN* rs1126772	AA AG + GG	108/151 66/89	1 1.07 (0.78–1.45)	0.170	1 1.06 (0.77–1.47)	0.720
	*OPN* rs11730582	TT TC + CC	54/64 121/177	1 0.66 (0.48–0.92)	**0.013**	1 0.67 (0.48–0.93)	**0.017**
	*CD44* rs187116	GG + GA AA	136/192 39/50	1 1.06 (0.74–1.51)	0.768	1 0.99 (0.67–1.45)	0.949
	*CD44* rs13347	CC CT + TT	116/160 59/82	1 0.93 (0.68–1.27)	0.642	1 0.95 (0.68–1.32)	0.758
	*CD44* rs7116432	AA AG + GG	59/77 116/165	1 0.87 (0.63–1.19)	0.382	1 1.02 (0.73–1.43)	0.896
	OPN levels	Low High	75/121 100/121	1 1.69 (1.25–2.28)	**<0.001**	1 1.54 (1.12–2.12)	**0.008**
LRFS	*OPN* rs4754	TT TC + CC	45/128 38/114	1 0.96 (0.63–1.49)	0.871	1 0.89 (0.56–1.41)	0.624
	*OPN* rs1126772	AA AG + GG	50/151 32/89	1 1.10 (0.71–1.72)	0.665	1 1.03 (0.64–1.63)	0.913
	*OPN* rs11730582	TT + TC CC	68/190 15/51	1 0.77 (0.44–1.35)	0.358	1 0.67 (0.37–1.20)	0.175
	*CD44* rs187116	GG + GA AA	70/192 13/50	1 0.70 (0.39–1.27)	0.237	1 0.78 (0.42–1.44)	0.425
	*CD44* rs13347	CC CT + TT	57/160 26/82	1 0.88 (0.55–1.39)	0.580	1 0.81 (0.50–1.31)	0.386
	*CD44* rs7116432	AA AG + GG	28/77 55/165	1 0.91 (0.58–1.43)	0.677	1 1.14 (0.71–1.84)	0.592
	OPN levels	Low High	39/121 44/121	1 1.26 (0.82–1.94)	0.293	1 1.18 (0.74–1.86)	0.489
MFS	*OPN* rs4754	TT TC + CC	19/128 13/114	1 0.80 (0.39–1.61)	0.526	1 0.78 (90.37–1.64)	0.514
	*OPN* rs1126772	AA AG + GG	24/151 8/89	1 0.57 (0.26–1.27)	0.170	1 0.54 (0.24–1.25)	0.147
	*OPN* rs11730582	TT TC + CC	11/64 21/177	1 0.63 (0.30–1.31)	0.214	1 0.57 (0.27–1.21)	0.144
	*CD44* rs187116	GG + GA AA	27/192 5/50	1 0.71 (0.27–1.83)	0.474	1 0.67 (0.25–1.86)	0.446
	*CD44* rs13347	CC CT + TT	24/160 8/82	1 0.61 (0.27–1.36)	0.227	1 0.41 (0.17–0.99)	**0.047**
	*CD44* rs7116432	AA + AG GG	24/201 8/41	1 1.82 (0.82–4.05)	0.144	1 2.11 (0.92–4.88)	0.079
	OPN levels	Low High	15/121 17/121	1 1.28 (0.64–2.57)	0.482	1 1.05 (0.50–2.21)	0.903
RT + CT subgroup (*n* = 123)							
OS	*OPN* rs4754	TT TC + CC	51/68 41/55	1 1.05 (0.69–1.58)	0.834	1 1.08 (0.70–1.66)	0.733
	*OPN* rs1126772	AA AG + GG	60/79 31/43	1 0.96 (0.62–1.48)	0.844	1 1.00 (0.64–1.56)	0.987
	*OPN* rs11730582	TT TC + CC	29/31 63/91	1 0.55 (0.36–0.86)	**0.009**	1 0.60 (0.38–0.96)	**0.032**
	*CD44* rs187116	GG + GA AA	74/103 18/20	1 1.43 (0.85–2.39)	0.177	1 1.03 (0.57–1.87)	0.923
	*CD44* rs13347	CC CT + TT	57/75 35/48	1 0.78 (0.51–1.19)	0.243	1 0.75 (0.47–1.19)	0.221
	*CD44* rs7116432	AA AG + GG	38/44 54/79	1 0.73 (0.48–1.10)	0.133	1 0.90 (0.58–1.40)	0.642
	OPN levels	Low High	37/61 55/62	1 1.99 (1.31–3.03)	**0.001**	1 1.66 (1.04–2.66)	**0.035**
LRFS	*OPN* rs4754	TT TC + CC	31/68 21/55	1 0.84 (0.48–1.46)	0.534	1 0.82 (0.47–1.46)	0.506
	*OPN* rs1126772	AA AG + GG	34/79 17/43	1 0.91 (0.51–1.63)	0.745	1 0.93 (0.51–1.69)	0.802
	*OPN* rs11730582	TT + TC CC	44/92 8/30	1 0.51 (0.24–1.08)	0.080	1 0.44 (0.20–0.96)	**0.040**
	*CD44* rs187116	GG GA + AA	16/36 36/87	1 0.90 (0.50–1.62)	0.724	1 0.91 (0.49–1.69)	0.774
	*CD44* rs13347	CC CT + TT	36/75 16/48	1 0.61 (0.34–1.10)	0.103	1 0.62 (0.33–1.15)	0.128
	*CD44* rs7116432	AA AG + GG	21/44 31/79	1 0.78 (0.45–1.36)	0.381	1 0.88 (0.50–1.57)	0.672
	OPN levels	Low High	22/61 30/62	1 1.63 (0.94–2.84)	0.084	1 1.41 (0.77–2.56)	0.262
MFS	*OPN* rs4754	TT TC + CC	13/68 8/55	1 0.77 (0.32–1.84)	0.552	1 0.69 (0.27–1.75)	0.434
	*OPN* rs1126772	AA AG + GG	17/79 4/43	1 0.42 (0.14–1.25)	0.119	1 0.37 (0.12–1.14)	0.084
	*OPN* rs11730582	TT TC + CC	8/31 13/91	1 0.46 (0.19–1.12)	0.089	1 0.46 (0.18–1.20)	0.111
	*CD44* rs187116	GG GA + AA	9/36 10/67	1 0.53 (0.22–1.26)	0.151	1 0.56 (0.22–1.40)	0.213
	*CD44* rs13347	CC CT + TT	16/75 5/48	1 0.40 (0.15–1.10)	0.075	1 0.23 (0.07–0.70)	**0.010**
	*CD44* rs7116432	AA AG + GG	10/44 11/79	1 0.59 (0.25–1.39)	0.225	1 0.80 (0.32–1.98)	0.623
	OPN levels	Low High	11/61 10/62	1 1.06 (0.45–2.50)	0.899	1 0.74 (0.27–2.05)	0.563
RT-only subgroup (*n* = 119)							
OS	*OPN* rs4754	TT TC + CC	41/60 42/59	1 1.07 (0.69–1.64)	0.773	1 1.16 (0.74–1.81)	0.521
	*OPN* rs1126772	AA AG + GG	48/72 35/46	1 1.20 (0.77–1.85)	0.421	1 1.21 (0.78–1.88)	0.406
	*OPN* rs11730582	TT + TC CC	70/98 13/21	1 0.68 (0.37–1.24)	0.209	1 0.62 (0.33–1.13)	0.118
	*CD44* rs187116	GG + GA AA	62/89 21/30	1 0.86 (0.52–1.42)	0.560	1 1.16 (0.67–2.02)	0.593
	*CD44* rs13347	CC CT + TT	59/85 24/34	1 1.09 (0.68–1.75)	0.728	1 1.18 (0.72–1.91)	0.510
	*CD44* rs7116432	AA AG + GG	21/33 62/86	1 1.16 (0.71–1.92)	0.548	1 1.22 (0.73–2.03)	0.456
	OPN levels	Low High	37/59 46/60	1 1.35 (0.88–2.09)	0.173	1 1.60 (0.98–2.62)	0.061
LRFS	*OPN* rs4754	TT TC + CC	14/60 17/59	1 1.31 (0.65–2.66)	0.454	1 1.60 (0.76–3.37)	0.218
	*OPN* rs1126772	AA AG + GG	16/72 15/46	1 1.56 (0.77–3.16)	0.215	1 1.63 (0.79–3.39)	0.188
	*OPN* rs11730582	TT + TC CC	24/98 7/21	1 1.33 (0.57–3.08)	0.509	1 1.33 (0.56–3.15)	0.520
	*CD44* rs187116	GG + GA AA	26/89 5/20	1 0.55 (0.21–1.43)	0.217	1 0.86 (0.31–2.42)	0.779
	*CD44* rs13347	CC CT + TT	21/85 10/34	1 1.28 (0.60–2.71)	0.526	1 1.76 (0.80–3.88)	0.165
	*CD44* rs7116432	AA AG + GG	7/33 24/86	1 1.39 (0.60–3.22)	0.445	1 1.48 (0.62–3.50)	0.378
	OPN levels	Low High	18/59 13/60	1 0.72 (0.35–1.46)	0.358	1 0.62 (0.30–1.28)	0.194
MFS	*OPN* rs4754	TT TC + CC	6/60 5/59	1 0.91 (0.28–3.00)	0.883	1 0.99 (0.29–3.35)	0.982
	*OPN* rs1126772	AA AG + GG	7/72 4/46	1 0.95 (0.28–3.23)	0.929	1 0.88 (0.25–3.05)	0.840
	*OPN* rs11730582	TT TC + CC	3/33 8/86	1 1.00 (0.26–3.77)	0.996	1 0.89 (0.23–3.40)	0.864
	*CD44* rs187116	GG GA + AA	1/32 10/87	1 3.75 (0.48–29.31)	0.208	1 8.89 (0.95–83.12)	0.055
	*CD44* rs13347	CC CT + TT	8/85 3/34	1 1.00 (0.26–3.75)	0.994	1 1.16 (0.30–4.48)	0.825
	*CD44* rs7116432	AA + AG GG	7/99 4/20	1 2.50 (0.73–8.55)	0.144	1 2.45 (0.66–9.11)	0.183
	OPN levels	Low High	4/59 7/60	1 1.84 (0.54–6.29)	0.330	1 2.07 (0.53–8.11)	0.294

uHR, univariable hazard ratio; aHR, multivariable hazard ratio form models including the respective molecular factor and all predefined clinico–demographic covariates; ^a^ numbers may not sum to the total due to missing genotype data for rs1126772 (*n* = 2) and for rs11730582 (*n* = 1); ^b^ model was adjusted for age at diagnosis, sex, T stage, N stage, tumor subsite, smoking and alcohol status, and CT use; *p* values ≤ 0.05 are shown in bold.

**Table 3:** Multivariable models for OS, LRFS, and MFS including all molecular and clinico-demographic factors in all patients and treatment subgroups.

Variable	OS		LRFS		MFS	
	HR (95% CI)	*p*	HR (95% CI)	*p*	HR (95% CI)	*p*
All patients (*n* = 242)						
Age (cont.)	1.02 (0.99–1.04)	0.073	1.01 (0.98–1.03)	0.722	1.01 (0.97–1.06)	0.579
Sex (male vs. female)	0.99 (0.63–1.55)	0.962	1.14 (0.61–2.16)	0.676	0.45 (0.18–1.15)	0.096
T stage (3–4 vs. 1–2)	1.23 (0.85–1.77)	0.269	1.34 (0.80–2.26)	0.267	0.91 (0.39–2.12)	0.821
N stage (1–3 vs. 0)	1.81 (1.16–2.83)	**0.010**	2.60 (1.32–5.12)	**0.006**	2.76 (0.89–8.51)	0.078
Smoking (ever vs. never)	1.37 (0.89–2.10)	0.148	1.30 (0.68–2.48)	0.421	1.26 (0.42–3.78)	0.682
Alcohol (ever vs. never)	1.34 (0.86–2.08)	0.197	1.33 (0.71–2.50)	0.367	3.07 (0.93–10.14)	0.066
OPSCC (yes vs. no)	1.32 (0.67–2.58)	0.424	1.54 (0.55–4.36)	0.411	0.31 (0.08–1.26)	0.101
HPSCC (yes vs. no)	2.04 (0.96–4.30)	0.062	2.42 (0.80–7.30)	0.116	1.28 (0.30–5.57)	0.738
LSCC (yes vs. no)	1.61 (0.77–3.36)	0.206	2.68 (0.88–8.17)	0.084	0.64 (0.14–2.95)	0.570
CT (yes vs. no)	1.11 (0.74–1.68)	0.610	1.41 (0.79–2.51)	0.240	1.65 (0.65–4.18)	0.289
*OPN* rs4754 (CC/TC vs. TT))	0.97 (0.55–1.69)	0.910	0.76 (0.34–1.72)	0.507	1.59 (0.48–5.25)	0.445
*OPN* rs1126772 (GG/AG vs. AA)	1.42 (0.77–2.65)	0.263	1.51 (0.66–3.48)	0.332	0.46 (0.11–1.85)	0.271
*OPN* rs11730582 (TC/CC vs. TT)	0.61 (0.41–0.91)	**0.015**	--	--	0.77 (0.31–1.96)	0.588
*OPN* rs11730582 (CC vs. TC/TT)	--	--	0.66 (0.36–1.22)	0.184	--	--
*CD44* rs187116 (AA vs. GG/GA)	0.96 (0.64–1.43)	0.830	0.72 (0.38–1.37)	0.316	0.67 (0.23–1.91)	0.450
*CD44* rs13347 (TT/CT vs. CC)	0.97 (0.68–1.37)	0.845	0.91 (0.54–1.52)	0.716	0.46 (0.18–1.13)	0.088
*CD44* rs7116432 (GG/AG vs. AA)	1.11 (0.77–1.59)	0.585	1.18 (0.71–1.95)	0.528	--	--
*CD44* rs7116432 (GG vs. AA/AG)	--	--	--	--	1.96 (0.80–4.80)	0.139
OPN levels (high vs. low)	1.62 (1.16–2.26)	**0.004**	1.20 (0.75–1.93)	0.443	1.12 (0.53–2.37)	0.774
RT + CT subgroup (*n* = 123)						
Age (cont.)	1.01 (0.98–1.04)	0.618	1.02 (0.98–1.06)	0.285	1.02 (0.96–1.08)	0.551
Sex (male vs. female)	1.29 (0.65–2.56)	0.459	1.26 (0.55–2.89)	0.592	0.82 (0.22–3.06)	0.771
T stage (3–4 vs. 1–2)	0.88 (0.53–1.46)	0.614	1.12 (0.56–2.23)	0.745	0.59 (0.18–1.91)	0.379
N stage (1–3 vs. 0)	1.49 (0.73–3.05)	0.276	1.08 (0.42–2.80)	0.864	2.22 (0.27–18.64)	0.461
Smoking (ever vs. never)	1.23 (0.69–2.21)	0.488	0.95 (0.45–2.00)	0.900	0.57 (0.14–2.26)	0.423
Alcohol (ever vs. never)	1.58 (0.81–3.09)	0.182	1.40 (0.59–3.36)	0.444	5.81 (0.98–34.30)	0.052
OPSCC (yes vs. no)	1.21 (0.59–2.47)	0.599	1.52 (0.54–4.30)	0.433	0.24 (0.05–1.18)	0.080
HPSCC (yes vs. no)	2.19 (0.98–4.92)	0.057	2.17 (0.69–6.84)	0.186	2.10 (0.45–9.68)	0.342
LSCC (yes vs. no)	1.71 (0.72–4.08)	0.227	1.60 (0.47–5.46)	0.456	0.34 (0.05–2.41)	0.277
*OPN* rs4754 (TC/CC vs. TT)	1.05 (0.49–2.24)	0.908	0.77 (0.26–2.28)	0.638	1.73 (0.33–9.19)	0.520
*OPN* rs1126772 (AG/GG vs. AA)	1.39 (0.58–3.34)	0.463	1.50 (0.48–4.69)	0.483	0.25 (0.03–1.87)	0.178
*OPN* rs11730582 (TC/CC vs. TT)	0.60 (0.34–1.05)	0.076	--	--	0.87 (0.25–3.00)	0.827
*OPN* rs11730582 (CC vs. TC/TT)	--	--	0.47 (0.20–1.09)	0.078	--	--
*CD44* rs187116 (AA vs. GG/GA)	0.91 (0.46–1.77)	0.771	--	--	--	--
*CD44* rs187116 (AA/GA vs. GG)	--	--	1.01 (0.52–1.98)	0.969	0.94 (0.31–2.87)	0.909
*CD44* rs13347 (CT/TT vs. CC)	0.71 (0.43–1.17)	0.181	0.63 (0.31–1.25)	0.184	0.19 (0.06–0.66)	**0.009**
*CD44* rs7116432 (GG/AG vs. AA)	0.94 (0.57–1.55)	0.809	0.80 (0.41–1.55)	0.503	0.41 (0.12–1.41)	0.156
OPN levels (high vs. low)	1.93 (1.15–3.23)	**0.013**	1.47 (0.78–2.76)	0.229	0.48 (0.17–1.41)	0.181
RT-only subgroup (*n* = 119)						
Age (cont.)	1.02 (0.98–1.06)	0.322	0.96 (0.90–1.01)	0.129	n.e.	n.e.
Sex (male vs. female)	0.80 (0.42–1.53)	0.504	1.18 (0.39–3.62)	0.776	n.e.	n.e.
T stage (3–4 vs. 1–2)	1.50 (0.87–2.57)	0.142	1.38 (0.59–3.25)	0.457	n.e.	n.e.
N stage (1–3 vs. 0)	2.32 (1.19–4.52)	**0.014**	6.00 (2.09–17.24)	**<0.001**	n.e.	n.e.
Smoking (ever vs. never)	1.61 (0.81–3.19)	0.177	2.53 (0.50–12.89)	0.263	n.e.	n.e.
Alcohol (ever vs. never)	0.97 (0.51–1.84)	0.929	1.10 (0.39–3.08)	0.856	n.e.	n.e.
OPSCC (yes vs. no)	0.80 (0.33–1.90)	0.608	0.37 (0.09–1.53)	0.170	n.e.	n.e.
LSCC (yes vs. no)	1.02 (0.41–2.55)	0.962	1.19 (0.32–4.46)	0.795	n.e.	n.e.
*OPN* rs4754 (TC/CC vs. TT))	0.98 (0.44–2.17)	0.958	1.07 (0.27–4.24)	0.926	n.e.	n.e.
*OPN* rs1126772 (AG/GG vs. AA)	1.58 (0.69–3.62)	0.280	1.13 (0.28–4.53)	0.861	n.e.	n.e.
*OPN* rs11730582 (CC vs. TC/TT)	0.43 (0.21–0.89)	**0.023**	0.87 (0.30–2.48)	0.789	n.e.	n.e.
*CD44* rs187116 (AA vs. GG/GA)	1.02 (0.57–1.83)	0.939	0.94 (0.31–2.84)	0.918	n.e.	n.e.
*CD44* rs13347 (CT/TT vs. CC)	1.30 (0.77–2.21)	0.325	1.85 (0.76–4.54)	0.176	n.e.	n.e.
*CD44* rs7116432 (GG/AG vs. AA)	1.25 (0.73–2.17)	0.417	2.08 (0.81–5.39)	0.130	n.e.	n.e.
OPN levels (high vs. low)	1.31 (0.81–2.11)	0.272	0.57 (0.25–1.27)	0.168	n.e.	n.e.

HR, hazard ratio for multivariable model; n.e., not estimated due to low number of events; *p* values ≤ 0.05 are shown in bold.

## Data Availability

The data supporting the findings of this study are included in the article and its Supplementary Materials. Due to ethical restrictions and the lack of patient consent for public data sharing, the datasets analyzed during the current study may be obtained from the corresponding author upon reasonable request and with appropriate ethical approval.

## Supplementary Materials

The following supporting information can be downloaded at: https://www.mdpi.com/article/10.3390/ijms27093724/s1.
