# Supplementary material for: Associations Between OPN-CD44 Axis Genetic Variability, Plasma Osteopontin, and Treatment Outcomes in Head and Neck Squamous Cell Carcinoma
Source: Int J Mol Sci. 2026 Apr 22;27(9):3724. doi: 10.3390/ijms27093724 (PMC13164495; doi:10.3390/ijms27093724)

Associations Between OPN-CD44 Axis Genetic Variability, Plasma Osteopontin, and Treatment Outcomes in Head and Neck Squamous Cell Carcinoma

Agnieszka Gdowicz-Kłosok, Regina Deja, Tomasz Rutkowski, Magdalena Bugowska, Jolanta Mrochem-Kwarciak, Krzysztof Składowski, and Dorota Butkiewicz

Supplementary Table S1. Genetic polymorphisms studied and their frequencies in the HNSCC patients.

SNP ID	Allele	Region	Function class [a]	Genotype distribution [b]	MAF	EU MAF [c]	HWE *p*
**SNPs in the *OPN* gene**							
rs1126772	A>G	3′ UTR	miRNA	151/81/8	0.20	0.21 (G)	0.471
rs11730582	T>C	promoter	TFBS	64/126/51	0.47	0.46 (C)	0.450
rs4754	T>C (Asp80Asp)	exon 5	splicing (ESE/ESS)	128/95/19	0.27	0.28 (C)	0.815
**SNPs in the *CD44* gene**							
rs187116	G>A	intron 1	---	68/124/50	0.46	0.46 (A)	0.635
rs13347	C>T	3′UTR	miRNA	160/73/9	0.19	0.22 (T)	0.851
rs7116432	A>G	3′UTR	miRNA	77/124/41	0.43	0.33 (G)	0.455

MAF, minor allele frequency in the studied group; HWE, Hardy-Weinberg equilibrium in the group; TFBS, transcription factor binding site; ESE/ESS, exonic splicing enhancer/exonic splicing silencer; [a] according to https://snpinfo.niehs.nih.gov; [b] shown in order of common homozygote, heterozygote, variant homozygote (missing data for rs1126772 in two patients and rs11730582 in one patient are not shown); [c] MAF in European population according to www.ensembl.org.

Supplementary Table S2. Individual SNPs and OPN levels in relation to OS, LRFS and MFS in all HNSCC patients and treatment subgroups with FDR correction.

Endpoint	Variable	Category	Events/*n* [a]	uHR (95% CI)	*p*	FDR	aHR (95% CI) [b]	*p*	FDR
All patients (*n* = 242)									
OS	*OPN* rs4754	TT	92/128	1			1		
		TC + CC	83/114	1.04 (0.77–1.41)	0.756	0.768	1.10 (0.80–1.50)	0.559	0.949
	OPN rs1126772	AA	108/151	1			1		
		AG + GG	66/89	1.07 (0.78–1.45)	0.170	0.397	1.06 (0.77–1.47)	0.720	0.949
	OPN rs11730582	TT	54/64	1			1		
		TC + CC	121/177	0.66 (0.48–0.92)	**0.013**	**0.045**	0.67 (0.48–0.93)	**0.017**	0.060
	CD44 rs187116	GG + GA	136/192	1			1		
		AA	39/50	1.06 (0.74–1.51)	0.768	0.768	0.99 (0.67–1.45)	0.949	0.949
	CD44 rs13347	CC	116/160	1			1		
		CT + TT	59/82	0.93 (0.68–1.27)	0.642	0.768	0.95 (0.68–1.32)	0.758	0.949
	CD44 rs7116432	AA	59/77	1			1		
		AG + GG	116/165	0.87 (0.63–1.19)	0.382	0.668	1.02 (0.73–1.43)	0.896	0.949
	OPN levels	Low	75/121	1			1		
		High	100/121	1.69 (1.25–2.28)	**< 0.001**	**0.005**	1.54 (1.12–2.12)	**0.008**	0.056
LRFS	*OPN* rs4754	TT	45/128	1			1		
		TC + CC	38/114	0.96 (0.63–1.49)	0.871	0.871	0.89 (0.56–1.41)	0.624	0.728
	OPN rs1126772	AA	50/151	1			1		
		AG + GG	32/89	1.10 (0.71–1.72)	0.665	0.789	1.03 (0.64–1.63)	0.913	0.913

	OPN rs11730582	TT + TC	68/190	1			1		
		CC	15/51	0.77 (0.44–1.35)	0.358	0.789	0.67 (0.37–1.20)	0.175	0.728
	CD44 rs187116	GG + GA	70/192	1			1		
		AA	13/50	0.70 (0.39–1.27)	0.237	0.789	0.78 (0.42–1.44)	0.425	0.728
	CD44 rs13347	CC	57/160	1			1		
		CT + TT	26/82	0.88 (0.55–1.39)	0.580	0.789	0.81 (0.50–1.31)	0.386	0.728
	CD44 rs7116432	AA	28/77	1			1		
		AG + GG	55/165	0.91 (0.58–1.43)	0.677	0.789	1.14 (0.71–1.84)	0.592	0.728
	OPN levels	Low	39/121	1			1		
		High	44/121	1.26 (0.82–1.94)	0.293	0.789	1.18 (0.74–1.86)	0.489	0.728
MFS	*OPN* rs4754	TT	19/128	1			1		
		TC + CC	13/114	0.80 (0.39–1.61)	0.526	0.526	0.78 (90.37–1.64)	0.514	0.600
	OPN rs1126772	AA	24/151	1			1		
		AG + GG	8/89	0.57 (0.26–1.27)	0.170	0.397	0.54 (0.24–1.25)	0.147	0.257
	OPN rs11730582	TT	11/64	1			1		
		TC + CC	21/177	0.63 (0.30–1.31)	0.214	0.397	0.57 (0.27–1.21)	0.144	0.257
	CD44 rs187116	GG + GA	27/192	1			1		
		AA	5/50	0.71 (0.27–1.83)	0.474	0.526	0.67 (0.25–1.86)	0.446	0.600
	CD44 rs13347	CC	24/160	1			1		
		CT + TT	8/82	0.61 (0.27–1.36)	0.227	0.397	0.41 (0.17–0.99)	**0.047**	0.257
	CD44 rs7116432	AA + AG	24/201	1			1		
		GG	8/41	1.82 (0.82–4.05)	0.144	0.397	2.11 (0.92–4.88)	0.079	0.257
	OPN levels	Low	15/121	1			1		
		High	17/121	1.28 (0.64–2.57)	0.482	0.526	1.05 (0.50–2.21)	0.903	0.903
RT + CT subgroup (*n* = 123)									
OS	*OPN* rs4754	TT	51/68	1			1		
		TC + CC	41/55	1.05 (0.69–1.58)	0.834	0.844	1.08 (0.70–1.66)	0.733	0.987
	OPN rs1126772	AA	60/79	1			1		
		AG + GG	31/43	0.96 (0.62–1.48)	0.844	0.844	1.00 (0.64–1.56)	0.987	0.987
	OPN rs11730582	TT	29/31	1			1		
		TC + CC	63/91	0.55 (0.36–0.86)	**0.009**	**0.031**	0.60 (0.38–0.96)	**0.032**	0.122
	CD44 rs187116	GG + GA	74/103	1			1		
		AA	18/20	1.43 (0.85–2.39)	0.177	0.310	1.03 (0.57–1.87)	0.923	0.987
	CD44 rs13347	CC	57/75	1			1		
		CT + TT	35/48	0.78 (0.51–1.19)	0.243	0.340	0.75 (0.47–1.19)	0.221	0.515
	CD44 rs7116432	AA	38/44	1			1		
		AG + GG	54/79	0.73 (0.48–1.10)	0.133	0.310	0.90 (0.58–1.40)	0.642	0.987
	OPN levels	Low	37/61	1			1		
		High	55/62	1.99 (1.31–3.03)	**0.001**	**0.007**	1.66 (1.04–2.66)	**0.035**	0.122
LRFS	*OPN* rs4754	TT	31/68	1			1		
		TC + CC	21/55	0.84 (0.48–1.46)	0.534	0.745	0.82 (0.47–1.46)	0.506	0.802
	OPN rs1126772	AA	34/79	1			1		
		AG + GG	17/43	0.91 (0.51–1.63)	0.745	0.745	0.93 (0.51–1.69)	0.802	0.802
	OPN rs11730582	TT + TC	44/92	1			1		
		CC	8/30	0.51 (0.24–1.08)	0.080	0.240	0.44 (0.20–0.96)	**0.040**	0.280
	CD44 rs187116	GG	16/36	1			1		
		GA + AA	36/87	0.90 (0.50–1.62)	0.724	0.745	0.91 (0.49–1.69)	0.774	0.802
	CD44 rs13347	CC	36/75	1			1		
		CT + TT	16/48	0.61 (0.34–1.10)	0.103	0.240	0.62 (0.33–1.15)	0.128	0.448
	CD44 rs7116432	AA	21/44	1			1		
		AG + GG	31/79	0.78 (0.45–1.36)	0.381	0.667	0.88 (0.50–1.57)	0.672	0.802
	OPN levels	Low	22/61	1			1		
		High	30/62	1.63 (0.94–2.84)	0.084	0.240	1.41 (0.77–2.56)	0.262	0.611

MFS	*OPN* rs4754	TT	13/68	1			1		
		TC + CC	8/55	0.77 (0.32–1.84)	0.552	0.644	0.69 (0.27–1.75)	0.434	0.608
	OPN	AA	17/79	1			1		
	rs1126772	AG + GG	4/43	0.42 (0.14–1.25)	0.119	0.264	0.37 (0.12–1.14)	0.084	0.259
	OPN	TT	8/31	1			1		
	rs11730582	TC + CC	13/91	0.46 (0.19–1.12)	0.089	0.264	0.46 (0.18–1.20)	0.111	0.259
	CD44	GG	9/36	1			1		
	rs187116	GA + AA	10/67	0.53 (0.22–1.26)	0.151	0.264	0.56 (0.22–1.40)	0.213	0.373
	CD44	CC	16/75	1			1		
	rs13347	CT + TT	5/48	0.40 (0.15–1.10)	0.075	0.264	0.23 (0.07–0.70)	**0.010**	0.070
	CD44	AA	10/44	1			1		
	rs7116432	AG + GG	11/79	0.59 (0.25–1.39)	0.225	0.315	0.80 (0.32–1.98)	0.623	0.623
	OPN levels	Low	11/61	1			1		
		High	10/62	1.06 (0.45–2.50)	0.899	0.899	0.74 (0.27–2.05)	0.563	0.623
RT-only ~~alone~~ subgroup (*n* = 119)									
OS	*OPN* rs4754	TT	41/60	1			1		
		TC + CC	42/59	1.07 (0.69–1.64)	0.773	0.773	1.16 (0.74–1.81)	0.521	0.593
	OPN	AA	48/72	1			1		
	rs1126772	AG + GG	35/46	1.20 (0.77–1.85)	0.421	0.773	1.21 (0.78–1.88)	0.406	0.593
	OPN	TT + TC	70/98	1			1		
	rs11730582	CC	13/21	0.68 (0.37–1.24)	0.209	0.731	0.62 (0.33–1.13)	0.118	0.413
	CD44	GG + GA	62/89	1			1		
	rs187116	AA	21/30	0.86 (0.52–1.42)	0.560	0.773	1.16 (0.67–2.02)	0.593	0.593
	CD44	CC	59/85	1			1		
	rs13347	CT + TT	24/34	1.09 (0.68–1.75)	0.728	0.773	1.18 (0.72–1.91)	0.510	0.593
	CD44	AA	21/33	1			1		
	rs7116432	AG + GG	62/86	1.16 (0.71–1.92)	0.548	0.773	1.22 (0.73–2.03)	0.456	0.593
	OPN levels	Low	37/59	1			1		
		High	46/60	1.35 (0.88–2.09)	0.173	0.731	1.60 (0.98–2.62)	0.061	0.413
LRFS	*OPN* rs4754	TT	14/60	1			1		
		TC + CC	17/59	1.31 (0.65–2.66)	0.454	0.526	1.60 (0.76–3.37)	0.218	0.381
	OPN	AA	16/72	1			1		
	rs1126772	AG + GG	15/46	1.56 (0.77–3.16)	0.215	0.526	1.63 (0.79–3.39)	0.188	0.381
	OPN	TT + TC	24/98	1			1		
	rs11730582	CC	7/21	1.33 (0.57–3.08)	0.509	0.526	1.33 (0.56–3.15)	0.520	0.607
	CD44	GG + GA	26/89	1			1		
	rs187116	AA	5/20	0.55 (0.21–1.43)	0.217	0.526	0.86 (0.31–2.42)	0.779	0.779
	CD44	CC	21/85	1			1		
	rs13347	CT + TT	10/34	1.28 (0.60–2.71)	0.526	0.526	1.76 (0.80–3.88)	0.165	0.381
	CD44	AA	7/33	1			1		
	rs7116432	AG + GG	24/86	1.39 (0.60–3.22)	0.445	0.526	1.48 (0.62–3.50)	0.378	0.529
	OPN levels	Low	18/59	1			1		
		High	13/60	0.72 (0.35–1.46)	0.358	0.526	0.62 (0.30–1.28)	0.194	0.381
MFS	*OPN* rs4754	TT	6/60	1			1		
		TC + CC	5/59	0.91 (0.28–3.00)	0.883	0.996	0.99 (0.29–3.35)	0.982	0.982
	OPN	AA	7/72	1			1		
	rs1126772	AG + GG	4/46	0.95 (0.28–3.23)	0.929	0.996	0.88 (0.25–3.05)	0.840	0.982
	OPN	TT	3/33	1			1		
	rs11730582	TC + CC	8/86	1.00 (0.26–3.77)	0.996	0.996	0.89 (0.23–3.40)	0.864	0.982
	CD44	GG	1/32	1			1		
	rs187116	GA + AA	10/87	3.75 (0.48–29.31)	0.208	0.728	8.89 (0.95–83.12)	0.055	0.385
	CD44	CC	8/85	1			1		
	rs13347	CT + TT	3/34	1.00 (0.26–3.75)	0.994	0.996	1.16 (0.30–4.48)	0.825	0.982
	CD44	AA + AG	7/99	1			1		
	rs7116432	GG	4/20	2.50 (0.73–8.55)	0.144	0.728	2.45 (0.66–9.11)	0.183	0.640

	OPN levels	Low<br>High	4/59<br>7/60	1<br>1.84 (0.54–6.29)	0.330	0.770	1<br>2.07 (0.53–8.11)	0.294	0.686

uHR, univariable hazard ratio; aHR, multivariable hazard ratio form models including the respective molecular factor and all predefined clinico-demographic covariates; FDR, *p* values adjusted using the Benjamini–Hochberg false discovery rate method; [a] Numbers numbers may not sum to the total due to missing genotype data for rs1126772 (*n* = 2), and for rs11730582 (*n* = 1); [b] Model model were adjusted for age at diagnosis, sex, T stage, N stage, tumor subsite, smoking and alcohol status, and CT use; *p* values ≤ 0.05 are shown in bold.

Supplementary Table S3. Univariable and multivariable analyses according to the *OPN* and *CD44* haplotypes for OS, LRFS and MFS in all HNSCC patients and treatment subgroups.

**SNPs**	**Endpoint**	**Haplotype**	**Number of copies**	**Events/ *n* [a]**	**uHR (95% CI)**	***p***	**FDR**	**aHR (95% CI) [b]**	***p***	**FDR**
				All patients (*n* = 242)						
OPN rs1173058-rs4754-rs1126772	OS	C-T-A	0<br>1–2	99/126<br>76/116	1<br>0.66 (0.49–0.90)	**0.007**	**0.035**	1<br>0.61 (0.44–0.84)	**0.002**	**0.010**
		T-T-A	0–1<br>2	138/199<br>37/43	1<br>1.49 (1.03–2.14)	**0.032**	0.080	1<br>1.56 (1.06–2.31)	**0.024**	0.060
		T-C-G	0<br>1	171/238<br>4/4	1<br>2.11 (0.78–5.72)	0.141	0.235	1<br>2.97 (1.03–8.55)	**0.044**	0.073
		C-C-G	0<br>1–2	113/157<br>62/85	1<br>1.02 (0.75–1.39)	0.901	0.901	1<br>1.01 (0.73–1.40)	0.936	0.936
		T-C-A	0<br>1–2	154/214<br>21/28	1<br>1.26 (0.80–1.99)	0.321	0.401	1<br>1.13 (0.70–1.83)	0.606	0.758
	LRFS	C-T-A	0<br>1–2	44/126<br>39/116	1<br>0.89 (0.58–1.37)	0.597	0.806	1<br>0.84 (0.54–1.31)	0.443	0.738
		T-T-A	0<br>1–2	18/70<br>65/172	1<br>1.57 (0.93–2.65)	0.090	0.450	1<br>1.85 (1.08–3.18)	**0.026**	0.130
		T-C-G	0<br>1	82/238<br>1/4	1<br>0.85 (0.12–6.09)	0.869	0.869	1<br>1.49 (0.19–11.32)	0.705	0.881
		C-C-G	0<br>1–2	52/157<br>31/85	1<br>1.11 (0.71–1.73)	0.645	0.806	1<br>1.01 (0.63–1.61)	0.976	0.976
		T-C-A	0<br>1–2	76/214<br>7/28	1<br>0.75 (0.34–1.62)	0.462	0.806	1<br>0.61 (0.27–1.34)	0.217	0.542
	MFS	C-T-A	0<br>1–2	17/126<br>15/116	1<br>0.87 (0.43–1.74)	0.695	0.869	1<br>0.79 (0.38–1.65)	0.530	0.530
		T-T-A	0<br>1–2	10/70<br>22/172	1<br>0.97 (0.46–2.06)	0.945	0.945	1<br>1.36 (0.53–3.47)	0.524	0.530
		T-C-G	0<br>1	31/238<br>1/4	1<br>2.10 (0.29–15.41)	0.465	0.775	1<br>7.27 (0.78–67.53)	0.081	0.210
		C-C-G	0<br>1–2	25/157<br>7/85	1<br>0.52 (0.22–1.20)	0.126	0.630	1<br>0.47 (0.20–1.11)	0.084	0.210
		T-C-A	0<br>1–2	27/214<br>5/28	1<br>1.61 (0.62–4.19)	0.327	0.775	1<br>1.44 (0.51–4.03)	0.490	0.530
CD44	OS	G-C	0<br>1–2	61/79<br>114/163	1<br>0.86 (0.63–1.17)	0.336	0.530	1<br>1.00 (0.72–1.40)	0.995	0.995

rs7116432-<br>rs13347		A-C	0<br>1–2	65/90<br>110/152	1<br>1.10 (0.81–1.50)	0.530	0.530	1<br>0.93 (0.68–1.28)	0.661	0.881
		A-T	0<br>1–2	118/162<br>57/80	1<br>0.89 (0.65–1.23)	0.487	0.530	1<br>0.91 (0.65–1.27)	0.579	0.881
		G-T	0<br>1–2	172/239<br>3/3	1<br>1.62 (0.52–5.09)	0.406	0.530	1<br>1.81 (0.55–5.92)	0.327	0.881
	LRFS	G-C	0<br>1–2	29/79<br>54/163	1<br>0.89 (0.57–1.40)	0.625	0.855	1<br>1.11 (0.69–1.79)	0.656	0.931
		A-C	0<br>1–2	29/90<br>54/152	1<br>1.11 (0.71–1.75)	0.641	0.855	1<br>1.02 (0.64–1.64)	0.931	0.931
		A-T	0<br>1–2	58/162<br>25/80	1<br>0.85 (0.53–1.36)	0.506	0.855	1<br>0.78 (0.47–1.27)	0.316	0.931
		G-T	0<br>1–2	82/239<br>1/31	1<br>0.98 (0.14–7.06)	0.986	0.986	1<br>1.19 (0.16–8.88)	0.867	0.931
	MFS	G-C	0<br>1–2	12/79<br>20/163	1<br>0.79 (0.39–1.62)	0.520	0.718	1<br>1.14 (0.53–2.46)	0.732	0.732
		A-C	0<br>1–2	13/90<br>19/152	1<br>0.88 (0.43–1.78)	0.718	0.718	1<br>0.72 (0.34–1.50)	0.374	0.561
		A-T	0<br>1–2	24/162<br>8/80	1<br>0.63 (0.28–1.40)	0.254	0.718	1<br>0.42 (0.17–1.01)	0.052	0.156
		G-T	0<br>1–2	32/239<br>0/3	n.e.	n.e.	n.e.	n.e.	n.e.	n.e.
RT + CT subgroup (*n* = 123)										
OPN<br>rs1173058-<br>rs4754-<br>rs1126772	OS	C-T-A	0<br>1–2	49/60<br>43/63	1<br>0.67 (0.44–1.01)	0.056	0.175	1<br>0.61 (0.39–0.95)	**0.029**	0.145
		T-T-A	0–1<br>2	74/103<br>18/20	1<br>1.52 (0.91–2.55)	0.112	0.187	1<br>1.46 (0.83–2.55)	0.188	0.313
		T-C-G	0<br>1	90/121<br>2/2	1<br>1.86 (0.45–7.60)	0.390	0.488	1<br>2.87 (0.62–13.16)	0.175	0.313
		C-C-G	0<br>1–2	63/82<br>29/41	1<br>0.90 (0.58–1.40)	0.651	0.651	1<br>0.92 (0.58–1.45)	0.720	0.720
		T-C-A	0<br>1–2	80/110<br>12/13	1<br>1.76 (0.96–3.24)	0.070	0.175	1<br>1.46 (0.73–2.89)	0.279	0.349
	LRFS	C-T-A	0<br>1–2	27/60<br>25/63	1<br>0.79 (0.46–1.36)	0.391	0.904	1<br>0.71 (0.40–1.25)	0.237	0.592
		T-T-A	0–1<br>2	42/103<br>10/20	1<br>2.11 (1.06–4.22)	**0.033**	0.165	1<br>2.41 (1.20–4.87)	**0.013**	0.065
		T-C-G	0<br>1	51/121<br>1/2	1<br>1.43 (1.20–10.39)	0.723	0.904	1<br>1.66 (0.20–13.52)	0.638	0.809
		C-C-G	0<br>1–2	36/82<br>16/41	1<br>0.86 (0.48–1.56)	0.625	0.904	1<br>0.87 (0.47–1.60)	0.653	0.809
		T-C-A	0<br>1–2	47/110<br>5/13	1<br>1.02 (0.41–2.58)	0.960	0.960	1<br>0.89 (0.33–2.37)	0.809	0.809
	MFS	C-T-A	0<br>1–2	11/60<br>10/63	1<br>0.74 (0.31–1.74)	0.485	0.493	1<br>0.77 (0.30–1.94)	0.573	0.764
		T-T-A	0<br>1–2	7/39<br>14/84	1<br>1.46 (0.49–4.36)	0.493	0.493	1<br>1.07 (0.41–2.83)	0.888	0.888
		T-C-G	0<br>1	21/121<br>0/2	n.e.	n.e.	n.e.	n.e.	n.e.	n.e.
		C-C-G	0<br>1–2	17/82<br>4/41	1<br>0.46 (0.16–1.37)	0.164	0.328	1<br>0.38 (0.12–1.17)	0.092	0.368

		T-C-A	0<br>1–2	17/110<br>4/13	1<br>2.32 (0.78–6.93)	0.130	0.328	1<br>2.25 (0.61–8.22)	0.221	0.442
CD44<br>rs7116432-<br>rs13347	OS	G-C	0<br>1–2	40/46<br>52/77	1<br>0.72 (0.47–1.08)	0.117	0.358	1<br>0.91 (0.59–1.43)	0.694	0.916
		A-C	0<br>1–2	31/46<br>61/77	1<br>1.23 (0.80–1.90)	0.342	0.456	1<br>0.98 (0.61–1.55)	0.916	0.916
		A-T	0<br>1–2	58/76<br>34/47	1<br>0.75 (0.49–1.14)	0.179	0.358	1<br>0.86 (0.53–1.41)	0.561	0.916
		G-T	0<br>1–2	90/121<br>2/2	1<br>1.25 (0.31–5.08)	0.758	0.758	1<br>1.44 (0.30–7.03)	0.650	0.916
	LRFS	G-C	0<br>1–2	22/46<br>30/77	1<br>0.78 (0.45–1.35)	0.370	0.493	1<br>0.88 (0.50–1.56)	0.656	0.875
		A-C	0<br>1–2	16/46<br>36/77	1<br>1.39 (0.77–2.51)	0.272	0.493	1<br>1.37 (0.74–2.55)	0.314	0.628
		A-T	0<br>1–2	37/76<br>15/47	1<br>0.57 (0.31–1.04)	0.066	0.264	1<br>0.56 (0.30–1.07)	0.078	0.312
		G-T	0<br>1–2	51/121<br>1/2	1<br>1.11 (0.15–8.07)	0.915	0.915	1<br>1.10 (0.13–9.21)	0.926	0.926
	MFS	G-C	0<br>1–2	10/46<br>11/77	1<br>0.63 (0.27–1.49)	0.292	0.428	1<br>0.81 (0.32–2.04)	0.654	0.654
		A-C	0<br>1–2	6/46<br>15/77	1<br>1.47 (0.57–3.79)	0.428	0.428	1<br>1.42 (0.50–4.08)	0.510	0.654
		A-T	0<br>1–2	16/76<br>5/47	1<br>0.41 (0.15–1.11)	0.079	0.237	1<br>0.23 (0.07–0.71)	**0.011**	**0.033**
		G-T	0<br>1–2	21/121<br>0/2	n.e.	n.e.	n.e.	n.e.	n.e.	n.e.
RT-only ~~alone~~ subgroup (*n* = 119)										
OPN<br>rs1173058-<br>rs4754-<br>rs1126772	OS	C-T-A	0<br>1–2	50/66<br>33/53	1<br>0.65 (0.42–1.01)	0.055	0.275	1<br>0.59 (0.37–0.93)	**0.024**	0.120
		T-T-A	0–1<br>2	18/31<br>65/88	1<br>1.48 (0.87–2.51)	0.145	0.362	1<br>1.59 (0.93–2.71)	0.091	0.152
		T-C-G	0<br>1	81/117<br>2/2	1<br>2.40 (0.59–9.80)	0.224	0.373	1<br>3.71 (0.82–16.85)	0.090	0.152
		C-C-G	0<br>1–2	50/75<br>33/44	1<br>1.16 (0.74–1.80)	0.517	0.646	1<br>1.15 (0.71–1.86)	0.582	0.728
		T-C-A	0<br>1–2	74/104<br>9/15	1<br>0.90 (0.45–1.80)	0.768	0.768	1<br>0.90 (0.44–1.86)	0.784	0.784
	LRFS	C-T-A	0<br>1–2	17/66<br>14/53	1<br>0.98 (0.48–2.00)	0.963	0.963	1<br>0.74 (0.34–1.62)	0.453	0.520
		T-T-A	0–1<br>2	8/31<br>23/88	1<br>1.23 (0.53–2.86)	0.628	0.837	1<br>1.69 (0.65–4.40)	0.281	0.520
		T-C-G	0<br>1	31/117<br>0/2	n.e.	n.e.	n.e.	n.e.	n.e.	n.e.
		C-C-G	0<br>1–2	16/75<br>15/44	1<br>1.70 (0.84–3.43)	0.141	0.564	1<br>1.40 (0.60–3.23)	0.433	0.520
		T-C-A	0<br>1–2	29/104<br>2/15	1<br>0.49 (0.12-2.06)	0.331	0.662	1<br>0.62 (0.14–2.68)	0.520	0.520
	MFS	C-T-A	0<br>1–2	6/66<br>5/53	1<br>0.99 (0.30–3.24)	0.982	0.982	1<br>0.82 (0.22–3.09)	0.770	0.990
		T-T-A	0–1<br>2	3/31<br>8/88	1<br>0.90 (0.19–4.20)	0.897	0.982	1<br>0.99 (0.19–5.09)	0.990	0.990
		T-C-G	0<br>1	10/117<br>1/2	1<br>7.88 (0.99–61.84)	0.052	0.260	1<br>30.38 (0.98–463.85)	0.058	0.290
		C-C-G	0	8/75	1			1		

			1–2	3/44	0.67 (0.18–2.52)	0.550	0.982	0.53 (0.12–2.21)	0.381	0.952
		T-C-A	0<br>1–2	10/104<br>1/15	1<br>0.77 (0.10–6.03)	0.805	0.982	1<br>0.78 (0.09–6.54)	0.820	0.990
CD44 rs7116432-rs13347	OS	G-C	0<br>1–2	21/33<br>62/86	1<br>1.16 (0.71–1.92)	0.548	0.940	1<br>1.20 (0.71–2.05)	0.494	0.538
		A-C	0<br>1–2	34/44<br>49/75	1<br>0.98 (0.63–1.53)	0.940	0.940	1<br>0.80 (0.51–1.28)	0.358	0.538
		A-T	0<br>1–2	60/86<br>23/33	1<br>1.05 (0.65–1.70)	0.846	0.940	1<br>1.18 (0.70–1.97)	0.538	0.538
		G-T	0<br>1	82/118<br>1/1	1<br>2.73 (0.37–19.92)	0.321	0.940	1<br>2.00 (0.23–17.29)	0.526	0.538
	LRFS	G-C	0<br>1–2	7/33<br>24/86	1<br>1.39 (0.60–3.22)	0.445	0.556	1<br>2.13 (0.84–5.40)	0.113	0.194
		A-C	0<br>1–2	13/44<br>18/75	1<br>0.81 (0.40–1.65)	0.556	0.556	1<br>0.61 (0.29–1.29)	0.194	0.194
		A-T	0<br>1–2	21/86<br>10/33	1<br>1.35 (0.63–2.84)	0.451	0.556	1<br>1.92 (0.82–4.50)	0.132	0.194
		G-T	0<br>1	31/118<br>0/1	n.e.	n.e.	n.e.	n.e.	n.e.	n.e.
	MFS	G-C	0<br>1–2	2/33<br>9/86	1<br>1.81 (0.39–8.38)	0.449	0.674	1<br>2.00 (0.41–9.73)	0.392	0.588
		A-C	0<br>1–2	7/44<br>4/75	1<br>0.35 (0.10–1.21)	0.097	0.291	1<br>0.30 (0.08–1.08)	0.065	0.195
		A-T	0<br>1–2	8/86<br>3/33	1<br>1.04 (0.28–3.93)	0.953	0.953	1<br>1.22 (0.29–5.20)	0.789	0.789
		G-T	0<br>1	11/118<br>0/1	n.e.	n.e.	n.e.	n.e.	n.e.	n.e.

uHR, univariate hazard ratio; aHR, adjusted hazard ratio for multivariable model without covariate preselection; FDR, p values adjusted using the Benjamini–Hochberg false discovery rate method; n.e., not estimated due to low number of events; [a] ~~Numbers~~ numbers may not sum to the total due to missing genotype data for rs1126772 in two patients, and for rs11730582 in one patient; [b] ~~Model~~ model adjusted for confounding factors such as age at diagnosis, sex, T stage, N stage, tumor subsite, smoking and alcohol status, and CT use; $p \leq 0.05$ shown in bold.

Supplementary Table S4. Pretreatment OPN levels in plasma (ng/mL) according to the genotypes.

**SNP**	***n* (%) [a]**	**OPN levels median (Q1–Q3)**	***p* [b]**
All patients, *n* = 242			
OPN rs4754			
TT	128 (53)	61.1 (15.7–90.3)	0.736
TC	95 (39)	63.2 (16.8–87.9)	
CC	19 (8)	42.2 (9.2–90.3)	
OPN rs1126772			
AA	151 (63)	61.6 (16.3–87.9)	0.303
AG	81 (34)	57.6 (16.1–94.2)	
GG	8 (3)	14.5 (8.8-63.8)	
OPN rs11730582			
TT	64 (27)	61.1 (16.6–86.6)	0.700
TC	126 (52)	63.8 (15.4–92.9)	
CC	51 (21)	52.7 (13.9–83.0)	
RT + CT subgroup (*n* = 123)			
OPN rs4754			
TT	68 (55)	63.9 (16.1–104.7)	0.556

TC	44 (36)	50.5 (15.0–104.7)	
CC	11 (9)	32.6 (9.2–81.4)	
OPN rs1126772			
AA	79 (65)	62.2 (16.3–106.6)	0.111
AG	40 (33)	50.5 (15.0–104.7)	
GG	3 (2)	9.2 (8.3–16.8)	
OPN rs11730582			
TT	31 (25)	61.6 (15.4–95.4)	0.709
TC	61 (50)	55.1 (15.9–110.2)	
CC	30 (25)	48.3 (13.9–82.2)	
RT~~-alone-~~only subgroup (*n* = 119)			
OPN rs4754			
TT	60 (50)	59.7 (14.8–84.7)	0.766
TC	51 (43)	68.0 (16.9–84.6)	
CC	8 (7)	57.5 (10.4–110.4)	
OPN rs1126772			
AA	72 (61)	61.1 (16.6–84.7)	0.821
AG	41 (35)	68.0 (16.8–84.6)	
GG	5 (4)	54.8 (12.2–72.8)	
OPN rs11730582			
TT	33 (28)	60.7 (17.9–86.3)	0.974
TC	65 (55)	64.4 (15.2–84.6)	
CC	21 (17)	62.4 (16.1–83.0)	

[a] Missing genotype data for rs1126772 in two patients, and for rs11730582 in one patient; [b] The Kruskall-Wallis H test; Q1, the first quartile; Q3, the third quartile.

Supplementary Table S5. Multivariable models for OS, LRFS, and MFS including all molecular and clinico-demographic factors in all patients and treatment subgroups with FDR correction.

Variable	OS			LRFS			MFS		
	HR (95% CI)	*p*	FDR	HR (95% CI)	*p*	FDR	HR (95% CI)	*p*	FDR
All patients (*n* = 242)									
Age (cont.)	1.02 (0.99–1.04)	0.073	0.248	1.01 (0.98–1.03)	0.722	0.722	1.01 (0.97–1.06)	0.579	0.769
Sex (male vs. female)	0.99 (0.63–1.55)	0.962	0.962	1.14 (0.61–2.16)	0.676	0.722	0.45 (0.18–1.15)	0.096	0.343
T stage (3–4 vs. 1–2)	1.23 (0.85–1.77)	0.269	0.457	1.34 (0.80–2.26)	0.267	0.628	0.91 (0.39–2.12)	0.821	0.821
N stage (1–3 vs. 0)	1.81 (1.16–2.83)	**0.010**	0.085	2.60 (1.32–5.12)	**0.006**	0.102	2.76 (0.89–8.51)	0.078	0.343
Smoking (ever vs. never)	1.37 (0.89–2.10)	0.148	0.419	1.30 (0.68–2.48)	0.421	0.628	1.26 (0.42–3.78)	0.682	0.821
Alcohol (ever vs. never)	1.34 (0.86–2.08)	0.197	0.438	1.33 (0.71–2.50)	0.367	0.628	3.07 (0.93–10.14)	0.066	0.343
OPSCC (yes vs. no)	1.32 (0.67–2.58)	0.424	0.655	1.54 (0.55–4.36)	0.411	0.628	0.31 (0.08–1.26)	0.101	0.343
HPSCC (yes vs. no)	2.04 (0.96–4.30)	0.062	0.248	2.42 (0.80–7.30)	0.116	0.628	1.28 (0.30–5.57)	0.738	0.821
LSCC (yes vs. no)	1.61 (0.77–3.36)	0.206	0.438	2.68 (0.88–8.17)	0.084	0.628	0.64 (0.14–2.95)	0.570	0.769
CT (yes vs. no)	1.11 (0.74–1.68)	0.610	0.798	1.41 (0.79–2.51)	0.240	0.628	1.65 (0.65–4.18)	0.289	0.614
OPN rs4754 (CC/TC vs. TT))	0.97 (0.55–1.69)	0.910	0.962	0.76 (0.34–1.72)	0.507	0.641	1.59 (0.48–5.25)	0.445	0.765
OPN rs1126772 (GG/AG vs. AA)	1.42 (0.77–2.65)	0.263	0.457	1.51 (0.66–3.48)	0.332	0.628	0.46 (0.11–1.85)	0.271	0.614

OPN rs11730582 (TC/CC vs. TT)	0.61 (0.41–0.91)	**0.015**	0.085	--	--	--	0.77 (0.31–1.96)	0.588	0.769
OPN rs11730582 (CC vs. TC/TT)	--	--	--	0.66 (0.36–1.22)	0.184	0.628	--	--	--
CD44 rs187116 (AA vs. GG/GA)	0.96 (0.64–1.43)	0.830	0.958	0.72 (0.38–1.37)	0.316	0.628	0.67 (0.23–1.91)	0.450	0.765
CD44 rs13347 (TT/CT vs. CC)	0.97 (0.68-1.37)	0.845	0.958	0.91 (0.54–1.52)	0.716	0.722	0.46 (0.18–1.13)	0.088	0.343
CD44 rs7116432 (GG/AG vs. AA)	1.11 (0.77–1.59)	0.585	0.798	1.18 (0.71–1.95)	0.528	0.641	--	--	--
CD44 rs7116432 (GG vs. AA/AG)	--	--	--	--	--	--	1.96 (0.80–4.80)	0.139	0.394
OPN levels (high vs. low)	1.62 (1.16–2.26)	**0.004**	0.068	1.20 (0.75–1.93)	0.443	0.628	1.12 (0.53–2.37)	0.774	0.821
RT + CT subgroup (*n* = 123)									
Age (cont.)	1.01 (0.98–1.04)	0.618	0.761	1.02 (0.98–1.06)	0.285	0.805	1.02 (0.96–1.08)	0.551	0.678
Sex (male vs. female)	1.29 (0.65–2.56)	0.459	0.761	1.26 (0.55–2.89)	0.592	0.851	0.82 (0.22–3.06)	0.771	0.881
T stage (3–4 vs. 1–2)	0.88 (0.53–1.46)	0.614	0.761	1.12 (0.56–2.23)	0.745	0.917	0.59 (0.18–1.91)	0.379	0.670
N stage (1–3 vs. 0)	1.49 (0.73–3.05)	0.276	0.631	1.08 (0.42–2.80)	0.864	0.960	2.22 (0.27–18.64)	0.461	0.670
Smoking (ever vs. never)	1.23 (0.69–2.21)	0.488	0.761	0.95 (0.45–2.00)	0.900	0.960	0.57 (0.14–2.26)	0.423	0.670
Alcohol (ever vs. never)	1.58 (0.81–3.09)	0.182	0.582	1.40 (0.59–3.36)	0.444	0.805	5.81 (0.98–34.30)	0.052	0.416
OPSCC (yes vs. no)	1.21 (0.59–2.47)	0.599	0.761	1.52 (0.54–4.30)	0.433	0.805	0.24 (0.05–1.18)	0.080	0.427
HPSCC (yes vs. no)	2.19 (0.98–4.92)	0.057	0.405	2.17 (0.69–6.84)	0.186	0.805	2.10 (0.45–9.68)	0.342	0.670
LSCC (yes vs. no)	1.71 (0.72–4.08)	0.227	0.605	1.60 (0.47–5.46)	0.456	0.805	0.34 (0.05–2.41)	0.277	0.633
OPN rs4754 (TC/CC vs. TT)	1.05 (0.49–2.24)	0.908	0.908	0.77 (0.26–2.28)	0.638	0.851	1.73 (0.33–9.19)	0.520	0.678
OPN rs1126772 (AG/GG vs. AA)	1.39 (0.58–3.34)	0.463	0.761	1.50 (0.48–4.69)	0.483	0.805	0.25 (0.03–1.87)	0.178	0.483
OPN rs11730582 (TC/CC vs. TT)	0.60 (0.34–1.05)	0.076	0.405	--	--	--	0.87 (0.25–3.00)	0.827	0.882
OPN rs11730582 (CC vs. TC/TT)	--	--	--	0.47 (0.20–1.09)	0.078	0.805	--	--	--
CD44 rs187116 (AA vs. GG/GA)	0.91 (0.46–1.77)	0.771	0.863	--	--	--	--	--	--
CD44 rs187116 (AA/GA vs. GG)	--	--	--	1.01 (0.52–1.98)	0.969	0.969	0.94 (0.31–2.87)	0.909	0.909
CD44 rs13347 (CT/TT vs. CC)	0.71 (0.43–1.17)	0.181	0.582	0.63 (0.31–1.25)	0.184	0.805	0.19 (0.06–0.66)	**0.009**	0.144
CD44 rs7116432 (GG/AG vs. AA)	0.94 (0.57–1.55)	0.809	0.863	0.80 (0.41–1.55)	0.503	0.805	0.41 (0.12–1.41)	0.156	0.483
OPN levels (high vs. low)	1.93 (1.15–3.23)	**0.013**	0.208	1.47 (0.78–2.76)	0.229	0.805	0.48 (0.17–1.41)	0.181	0.483
RT ~~alone~~ only subgroup (*n* = 119)									
Age (cont.)	1.02 (0.98–1.06)	0.322	0.609	0.96 (0.90–1.01)	0.129	0.440	n.e.	n.e.	n.e.
Sex (male vs. female)	0.80 (0.42–1.53)	0.504	0.756	1.18 (0.39–3.62)	0.776	0.926	n.e.	n.e.	n.e.
T stage (3–4 vs. 1–2)	1.50 (0.87–2.57)	0.142	0.609	1.38 (0.59–3.25)	0.457	0.857	n.e.	n.e.	n.e.
N stage (1–3 vs. 0)	2.32 (1.19–4.52)	**0.014**	0.172	6.00 (2.09–17.24)	**<0.001**	**0.013**	n.e.	n.e.	n.e.
Smoking (ever vs. never)	1.61 (0.81–3.19)	0.177	0.609	2.53 (0.50–12.89)	0.263	0.563	n.e.	n.e.	n.e.
Alcohol (ever vs. never)	0.97 (0.51–1.84)	0.929	0.962	1.10 (0.39–3.08)	0.856	0.926	n.e.	n.e.	n.e.

OPSCC (yes vs. no)	0.80 (0.33–1.90)	0.608	0.829	0.37 (0.09–1.53)	0.170	0.440	n.e.	n.e.	n.e.
LSCC (yes vs. no)	1.02 (0.41–2.55)	0.962	0.962	1.19 (0.32–4.46)	0.795	0.926	n.e.	n.e.	n.e.
OPN rs4754 (TC/CC vs. TT)	0.98 (0.44–2.17)	0.958	0.962	1.07 (0.27–4.24)	0.926	0.926	n.e.	n.e.	n.e.
OPN rs1126772 (AG/GG vs. AA)	1.58 (0.69–3.62)	0.280	0.609	1.13 (0.28–4.53)	0.861	0.926	n.e.	n.e.	n.e.
OPN rs11730582 (CC vs. TC/TT)	0.43 (0.21–0.89)	**0.023**	0.172	0.87 (0.30–2.48)	0.789	0.926	n.e.	n.e.	n.e.
CD44 rs187116 (AA vs. GG/GA)	1.02 (0.57–1.83)	0.939	0.962	0.94 (0.31–2.84)	0.918	0.926	n.e.	n.e.	n.e.
CD44 rs13347 (CT/TT vs. CC)	1.30 (0.77–2.21)	0.325	0.609	1.85 (0.76–4.54)	0.176	0.440	n.e.	n.e.	n.e.
CD44 rs7116432 (GG/AG vs. AA)	1.25 (0.73–2.17)	0.417	0.695	2.08 (0.81–5.39)	0.130	0.440	n.e.	n.e.	n.e.
OPN levels (high vs. low)	1.31 (0.81–2.11)	0.272	0.609	0.57 (0.25–1.27)	0.168	0.440	n.e.	n.e.	n.e.

HR, hazard ratio for multivariable model; FDR, *p* values adjusted using the Benjamini–Hochberg false discovery rate method; n.e., not estimated due to low number of events; *p* values ≤ 0.05 are shown in bold.

Supplementary Figure S1. Linkage disequilibrium (LD) analysis for (**a**) *OPN* rs11730582, rs4754 and rs1126772, and (**b**) *CD44* rs187116, rs7116432 and rs13347, including (**c**) the exact pairwise LD values among *OPN* and *CD44* polymorphisms. The color intensity indicates LD strength, and the numbers in the cells represent the pairwise D' values.

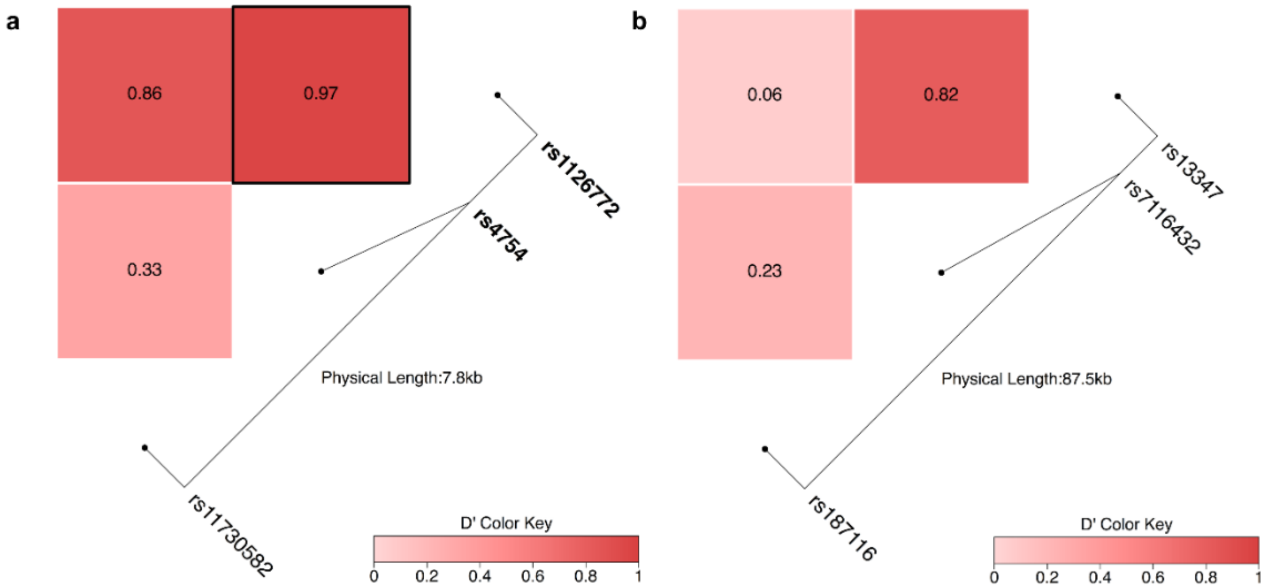

c

SNP 1	SNP 2	D' (95% CI)	r^2	X^2	p
		**Pairwise LD among *OPN* polymorphisms**			
rs11730582	rs4754	0.33 (0.21–0.45)	0.05	22.48	**<.001**
	rs1126772	0.86 (0.79–0.92)	0.21	99	**<.001**
rs4754	rs1126772	0.97 (0.94–1.00)	0.63	301.72	**<.001**
		**Pairwise LD among *CD44* polymorphisms**			
rs187116	rs7116432	0.23 (0.10–0.35)	0.04	21.37	**<.001**
	rs13347	0.06 (-0.06–0.18)	0.00	0.47	**<.001**
rs7116432	rs13347	0.82 (0.74–0.89)	0.11	55.23	**<.001**

Supplementary Figure S2. The Kaplan-Meier plots according to the *OPN* rs1173058-rs4754-rs1126772 haplotypes: (**a**) C-T-A and OS in all patients, (**b**) T-T-A and OS in all patients, and (**c**) T-T-A and LRFS in the combination treatment subgroup (RT + CT).

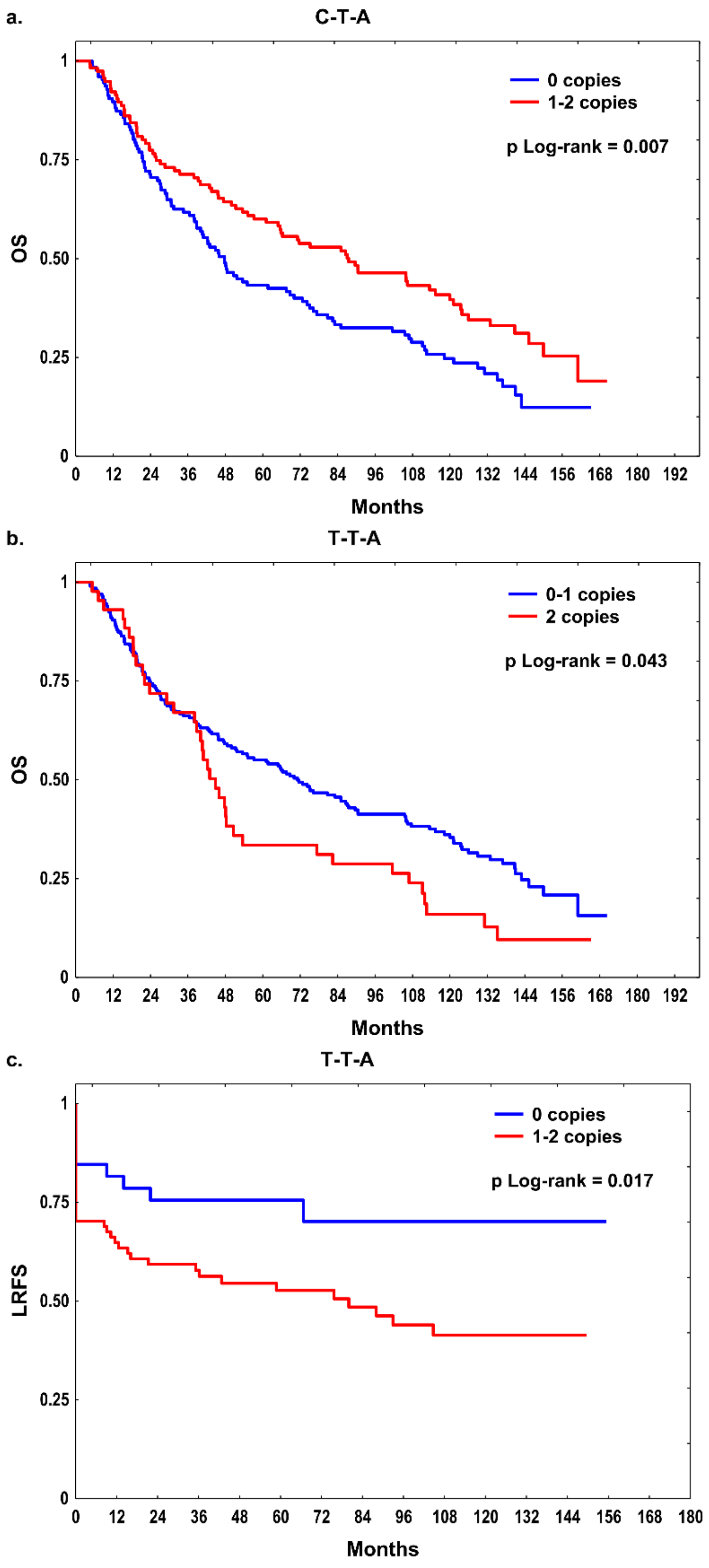

Supplement: Supplementary file 1 [file ijms-27-03724-s001.zip › ijms-4239118-supplementary.pdf]
